# Supplementary material for: An alternative pathway for membrane protein biogenesis at the endoplasmic reticulum
Source: Commun Biol. 2021 Jul 1;4:828. doi: 10.1038/s42003-021-02363-z (PMC8249459; doi:10.1038/s42003-021-02363-z)

Supplementary information for

An alternative pathway for membrane protein biogenesis at the endoplasmic reticulum

Sarah O'Keefe, Guanghui Zong, Kwabena B. Duah, Lauren E. Andrews, Wei Q. Shi and Stephen High

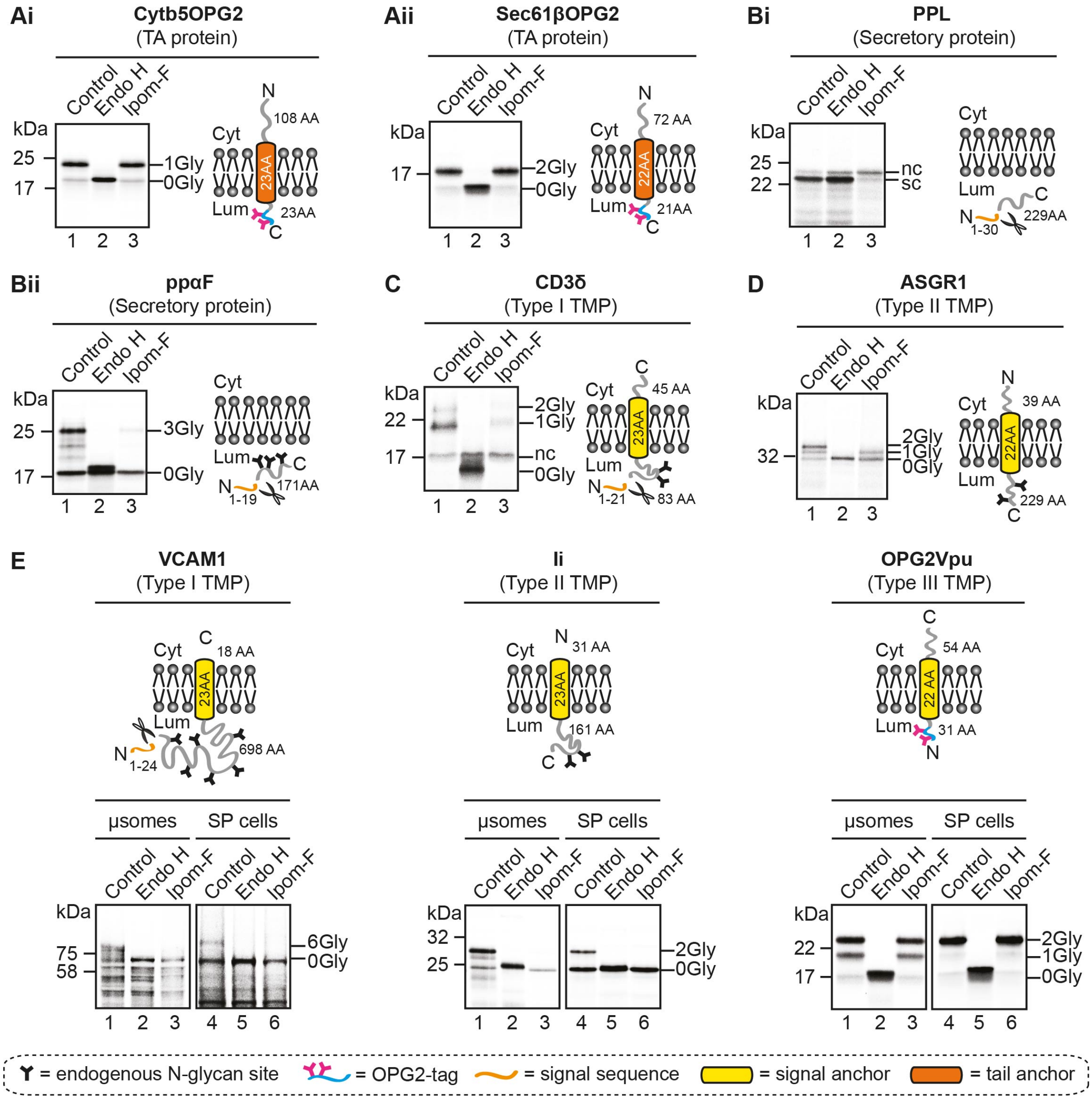

Supplementary Figure 1. The specificity of an Ipom-F induced translocational block is consistent and reproducible in ER-derived microsomes and SP cells, Related to Figures 1-6.

The tail-anchored protein precursors, (**Ai**) C-terminally OPG2-tagged cytochrome b5 (Cytb5OPG2) and (**Aii**) C-terminally OPG2-tagged Sec61 beta subunit (Sec61βOPG2); secretory proteins, (**Bi**) bovine preprolactin (PPL) and (**Bii**) yeast prepro-alpha-factor (ppαF); single-pass type I transmembrane protein (TMP), (**C**) T-cell surface glycoprotein CD3 delta chain (CD3δ) and single-pass type II TMP (**D**) asialoglycoprotein receptor 1 (ASGR1) were synthesised in rabbit reticulocyte lysate supplemented with ER-derived canine pancreatic microsomes in the presence and

absence of 1 μM Ipom-F. (**E**) The effect of 1 μM Ipom-F on insertion into both ER-derived microsomes and SP cells was determined using a model substrate from each class of single-pass TMP; the type I TMP, vascular cell adhesion protein 1 (VCAM1), the type II TMP, isoform 2 (residues 17-232) of the short form of HLA class II histocompatibility antigen gamma chain (Ii) and the type III TMP OPG2Vpu (as Fig. 1di). Phosphorimages of membrane-associated products resolved by SDS-PAGE together with representative substrate outlines are shown. N-glycosylation or signal sequence cleavage were used as a read-out for the qualitative efficiency of translocation/insertion. N-glycosylated (X-Gly) versus non-N-glycosylated (0Gly) species were identified by treatment with endoglycosidase H (Endo H, lane 2). Sc, signal cleaved form, nc, non-signal cleaved form.

A

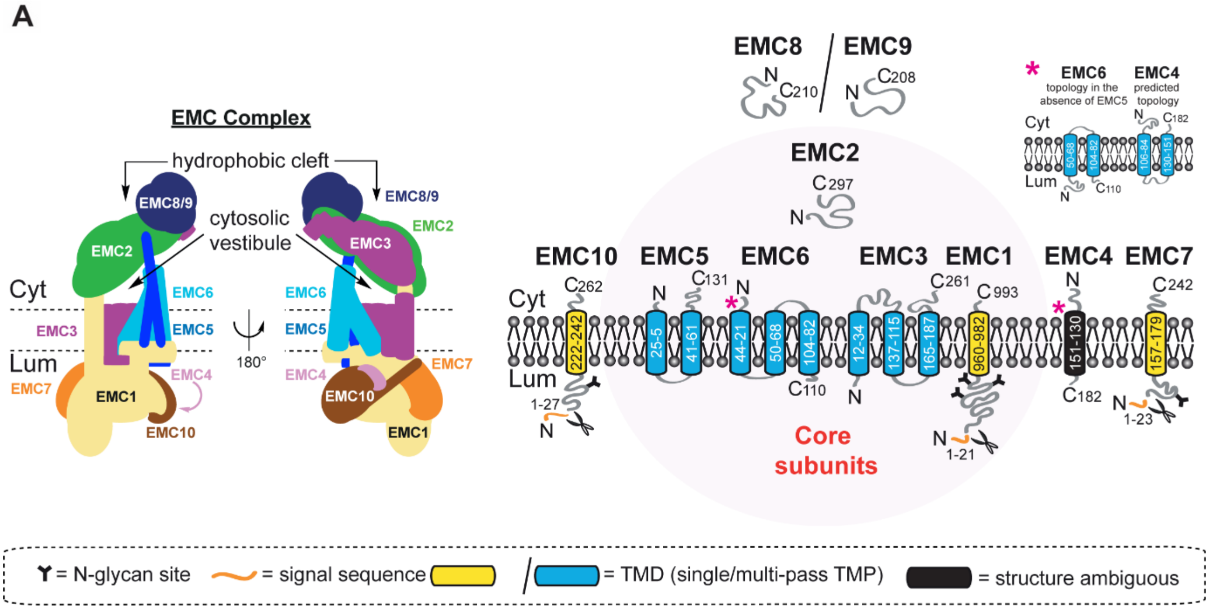

B

Subunit	Structurally integral?	Protein Type	Mw (kDa)	Human	Dog	Sequence identity
**EMC1**	Core subunit	Type I TMP	112	Q8N766	F1PN78	95.5%
**EMC2**	Core subunit	Cytosolic	35	Q15006	E2R0P8	100%
**EMC3**	Core subunit	Multi-pass TMP	30	QP012	E3RMQ3	99.5%
**EMC4**	X	Multi-pass TMP	20	Q5J8M3	E2R5T1	97.8%
**EMC5**	Core subunit	Multi-pass TMP	15	Q8N4V1	N/A	N/A
**EMC6**	Core subunit	Multi-pass TMP	12	Q8N766	E2R7Q9	99.1%
**EMC7**	X	Type I TMP	26	Q9NPA0	J6P692	95.5%
**EMC8**	X	Cytosolic	24	O4302	E2QUA5	98.6%
**EMC9**	X	Cytosolic	23	Q9Y3B6	F6UPE2	92.7%
**EMC10**	X	Type I TMP	27	Q5UCC4	E2R112	92.0%

C

```
                                TMD1
S. cerevisiae    1  MS-----------------------------------FVSKLLYTVSALVLFHSGFSSYEFHHLLKLNSL   35
O. aries         1  MAPSLWKGLVGIGLFALAHAAFSAAQHYFPSSGIKWKMKCEFLQSSSFQDKIFRSMYYVYDRSYMRLTEK   70
M. musculus      1  MAPSLWKGLVGVGLFALAHAAFSAAQH----------------------------------RSYMRLTEK   36
H. sapiens       1  MAPSLWKGLVGIGLFALAHAAFSAAQH----------------------------------RSYMRLTEK   36

                                TMD2
S. cerevisiae   36  NNAQGAISKLPKDIMYETYAGLILFVLAVFTSFEKLQYLPIESNDGKIISQGNYLKEIALNKATNVDNLI  105
O. aries        71  ED-----ESLPIDIVLQTLLAFAVTCYGIVHIAGEFKDMD----------ATSELKNK------------  113
M. musculus     37  ED-----ESLPIDIVLQTLLAFAVTCYGIVHIAGEFKDMD----------ATSELKNK------------   79
H. sapiens      37  ED-----ESLPIDIVLQTLLAFAVTCYGIVHIAGEFKDMD----------ATSELKNK------------   79

S. cerevisiae  106  GSNPNGEIIFTPSFVDVH---------------------------MKRKICREWASNTVKKEK  141
O. aries       114  ---TFDTLRNHPSFYVFNHRGRVLFRPSDTTNSSNQDALSSNTSLKLRKL--ESLRR------  165
M. musculus     80  ---TFDTLRNHPSFYVFNHRGRVLFRPSDATNSSNLDALSSNTSLKLRKF--DSLRR------  131
H. sapiens      80  ---TFDTLRNHPSFYVFNHRGRVLFRPSDTANSSNQDALSSNTSLKLRKL--ESLRR------  131
```

Supplementary Figure 2. The human EMC is a 9-subunit multimeric complex, Related to Fig. 2.

(**A**) A schematic of the structure of the human EMC, depicting its tripartite organisation across the ER membrane (a basket-shaped cytosolic region, an ordered membrane-spanning core and an L-shaped ER lumenal region)[1,2,3] and the topology of each of its subunits; three soluble/cytosolic (EMC2, EMC8, EMC9), three single-pass type I TMPs (EMC1, EMC7, EMC10), three multi-pass TMPs (EMC3, EMC5, EMC6) and one topologically ambiguous subunit whose N-terminus is cytosolic[3,4] but may be comprised of one[1,2,3], two[5] or three[2,3] transmembrane domains (EMC4). Of these 10 subunits, EMC8 and EMC9 are functional paralogues, thus the EMC complex may either be comprised of EMC1-8 or EMC1-7,9 together with EMC10[1,2,3]. In addition, EMC6 is topologically dependent on the presence of EMC5, whereby the poorly hydrophobic first transmembrane domain of EMC6 is only capable of inserting into the membrane upon assembly with EMC5[1]. EMC subunits with variable or an unconfirmed number of TMDs are indicated by (*). (**B**) Based on subunit depletion studies, and consistent with its ER membrane organisation, EMC1, EMC2, EMC3, EMC5, EMC6 are structurally integral to the assembly of the wider EMC complex[1,6]. Subunits EMC1-10, whether structurally integral or not, are highly conserved between humans and dogs, with the exception of EMC5 which is not present at all in dogs. (**C**) The structurally-integral subunit human EMC5 (*H. sapiens*, Q8N4V1-1) is conserved in yeast (*S. cerevisiae*, P40540, 22% identity, 54% similarity) and shares high homology with its sheep (*O. aries*, W5PPL4, 79% identity, 79% similarity) and mouse (*M. musculus*, Q8K273, 95% identity, 99% similarity) equivalents.

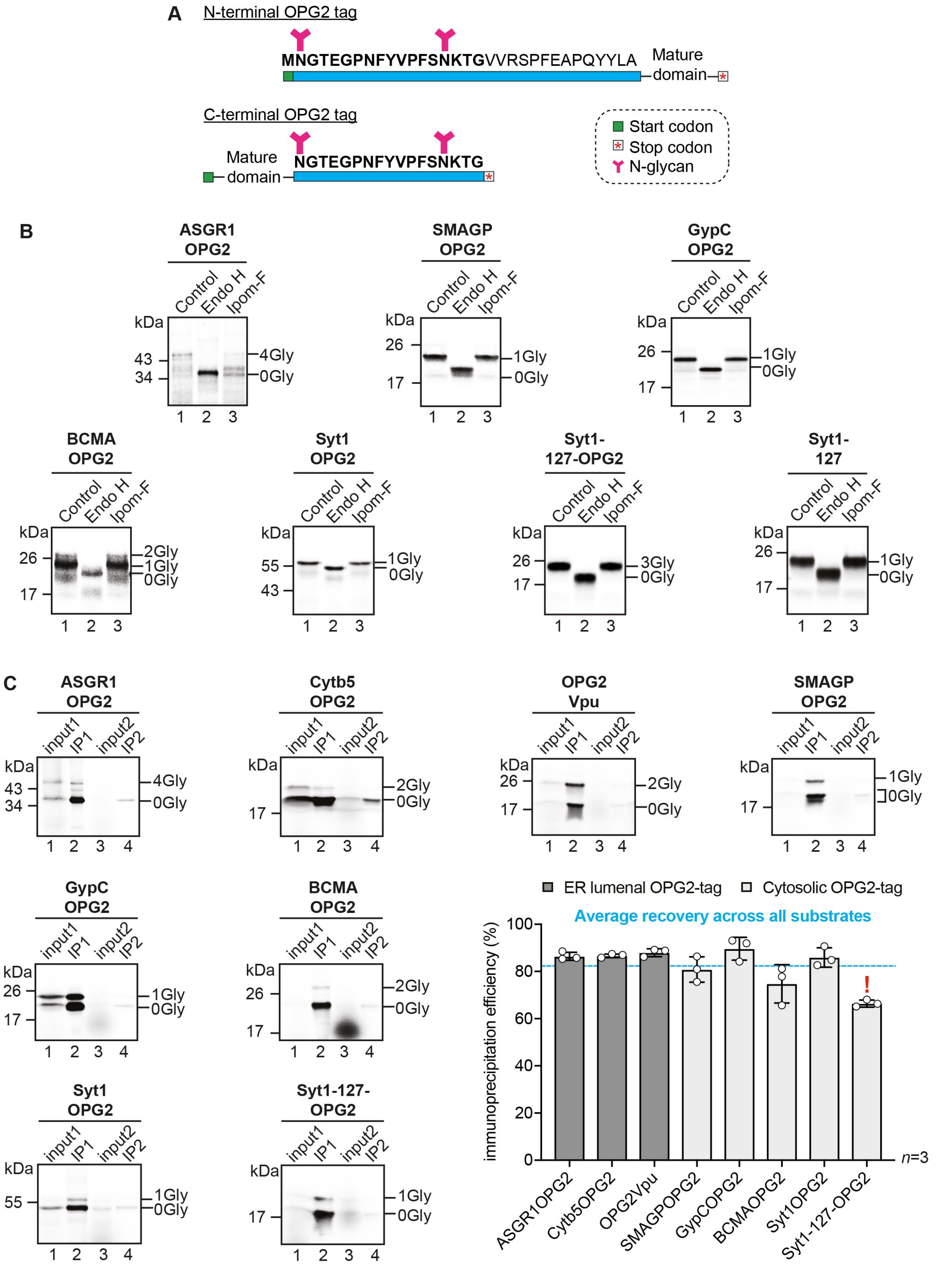

Supplementary Figure 3. The OPG2-tag does not affect Ipom-F sensitivity of TMPs and permits efficient recovery of TMP substrates by immunoprecipitation independent of OPG2-tag location, Related to Figures 1-2

and 4-6. (**A**) An N-terminal fragment of bovine rhodopsin containing two sites for N-glycosylation (OPG2-tag) was incorporated either at the N-terminus (residues 1-26 rhodopsin) or C-terminus (residues 2-18 rhodopsin) of model TMPs. (**B**) Phosphorimages of the membrane-associated radiolabelled products synthesised in ER-derived canine pancreatic microsomes. Precursor polypeptides include OPG2-tagged (**Bi**) asialoglycoprotein receptor 1 (ASGR1OPG2), (**Bii**) small cell adhesion glycoprotein containing an artificial N-glycan site (SMAGPOPG2), (**Biii**) glycophorin C (GypCOPG2), (**Biv**) tumour necrosis factor receptor superfamily member 17 containing an artificial N-glycan site (BCMAOPG2), (**Bv**) synaptotagmin 1 (Syt1OPG2), (**Bvi**) a truncated version of Syt1OPG2 (Syt1-127-OPG2) and (**Bvii**) a non-tagged version of Syt-127-OPG2 (Syt1-127). (**C**) Each of the OPG2-tagged precursor polypeptides used in this study were synthesised with semi-permeabilised cells, recovered via the OPG2-tag by immunoprecipitation (IP1; lanes 1 and 2) and analysed by SDS-PAGE and phosphorimaging. Any remaining proteins contained in the supernatant were then isolated by immunoprecipitating a second time (IP2; lanes 3 and 4). Inputs are 10% of the total material. The relative efficiencies of TMP recovery were calculated as a ratio of the total signal intensity present in IP1 compared to that in present in IP1+IP2. Quantifications are given as means ±SEM for three independent immunoprecipitations (n=3). (!) indicates a slightly reduced calculation of recovery due to the presence of radiolabelled material unrelated to the protein of interest (Syt1-127-OPG2, lane 3).

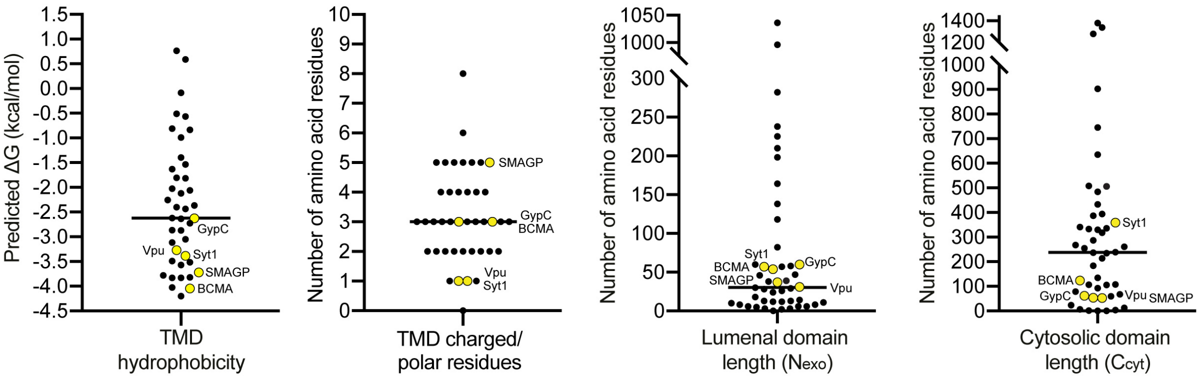

Supplementary Figure 4. Predicted hydrophobicity of transmembrane domains from human type III TMPs, Related to Figures 1-7.

Predicted transmembrane domain (TMD) hydrophobicity values (ΔG_{app})[5], the number of charged and/or polar amino acid residues in the transmembrane domain and lumenal/cytoplasmic (Nexo/Ccyt) domain lengths were calculated by inputting the protein sequences of 38 human, 1 rat (Syt1) and 1 viral (HIV-Vpu) type III TMPs (Uniprot annotated) into the 'full protein scan' mode using http://dgpred.cbr.su.se/. Type III TMPs analysed in this study are in yellow. All other Uniprot annotated type III TMPs that enter the secretory pathway (i.e. are localized in the ER, Golgi apparatus or plasma membrane) are in black. For a full list of type III TMPs analysed, and those discounted from analysis, see Supplementary Data 1. Charged amino acid residues, D, E, H, K, R; polar amino acid residues, N, Q, S, T, Y.

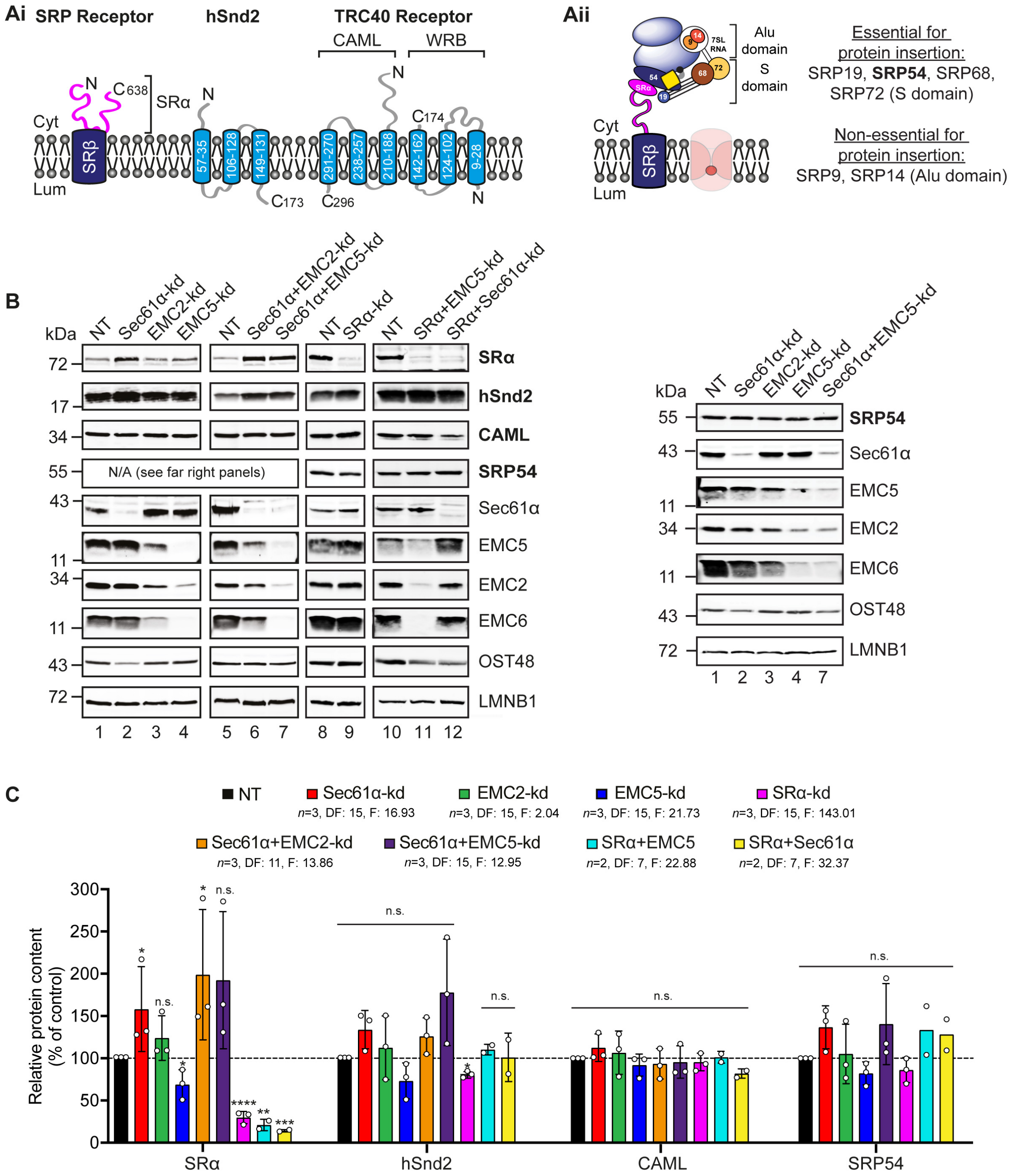

Supplementary Figure 5. Knockdowns of subunits of the Sec61, EMC and/or SRP receptor complexes variably affect the levels of ER protein translocation components, Related to Figures 2-6.

(**Ai**) Representative structures of known membrane-bound receptors involved in protein translocation. The SRP-(signal recognition particle) receptor (co-translational pathway) is a heterodimer comprised of the membrane-integrated beta-subunit (SRβ) which anchors the peripherally associated cytosolic alpha-subunit (SRα) to the

ER membrane. SRα binds to SRP and transfers SRP-targeted ribosome nascent chains to the Sec61 complex[7]. hSnd2 (co/post-translational pathway) is the human orthologue of a component involved in the distinct SRP-independent (SND) targeting pathway present in yeast[8]. hSnd2 plays a role in the ER targeting of certain proteins, although this pathway is yet to be fully defined in humans[9]. Here, we show hSnd2 as a TMP containing three transmembrane domains[5,10], although a four transmembrane domain model has also been hypothesised[9]. The TRC40 receptor (post-translational pathway), comprised of the WRB and CAML-(calcium signal-modulating cyclophilin ligand) membrane proteins, may either insert TRC40-targeted tail-anchor proteins into the ER membrane directly[11] or re-route certain proteins to the Sec61 complex[12]. (**Aii**) A schematic of SRP-(signal recognition particle) which is comprised of a highly base paired SRP RNA (7SL) and six protein subunits (SRP9, SRP14, SRP19, SRP54, SRP68 and SRP72) that are assembled into a functionally independent Alu domain (responsible for translational retardation) and the S domain (signal sequence engagement and SRP receptor binding). Each subunit of the S domain is essential for SRP function whereas those of the Alu domain are dispensable for protein translocation/insertion[7]. (**B**) The effect on SRα, hSnd2, CAML and SRP54 of transfecting HeLa cells with non-targeting (NT; lanes 1, 5, 8 and 10), Sec61α-targeting (lane 2), EMC2-targeting (lane 3), EMC5-targeting (lane 4), Sec61α+EMC2-targeting (lane 6), Sec61α+EMC5-targeting (lane 7), SRα-targeting (lane 9), SRα+EMC5-targeting (lane 11) and SRα+Sec61α-targeting (lane 12) siRNAs were determined after semi-permeabilisation by immunoblotting. (**C**) The effects of each siRNA-mediated knockdown were calculated as a proportion of the signal intensity obtained with the NT control (set as 100%). Quantifications are given as means ±SEM for two or three separate siRNA treatments (n=2 or n=3 as indicated in figure) with statistical significance of siRNA-mediated knockdowns (two-way ANOVA) determined using Sidak's multiple comparisons test. Statistical significance is given as n.s., non-significant; *, $P < 0.05$; **, $P < 0.01$; ***, $P < 0.001$; ****, $P < 0.0001$.

Supplementary Figure 6

Uncropped and unedited blot/gel images

Fig. 1d: Phosphorimages of +/- Ipom-F insertion assay in microsomes

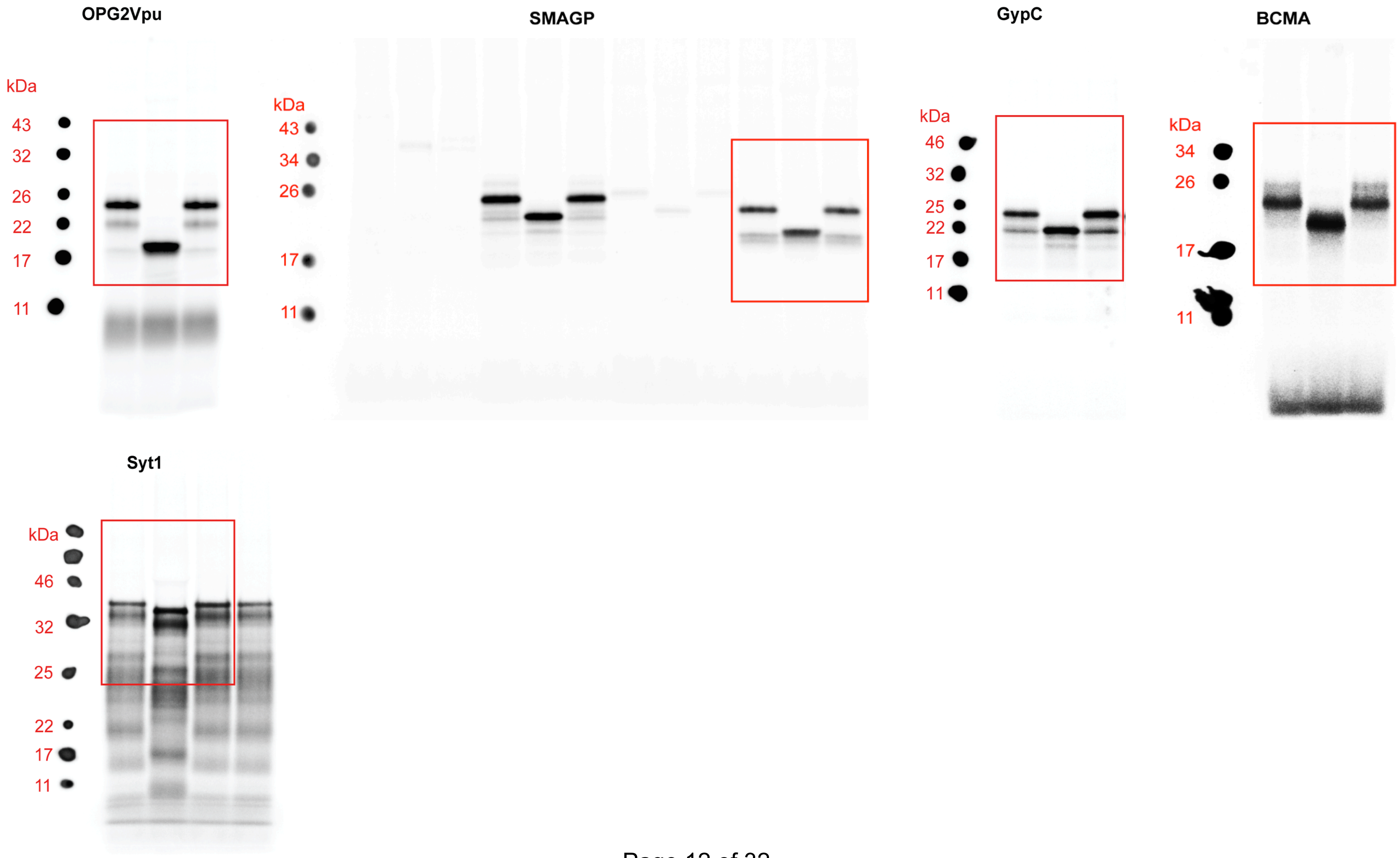

Fig. 2c: Efficiency of single knockdowns; NT, Se61α-kd, EMC2-kd, EMC5-kd

<u>SP Cells</u>

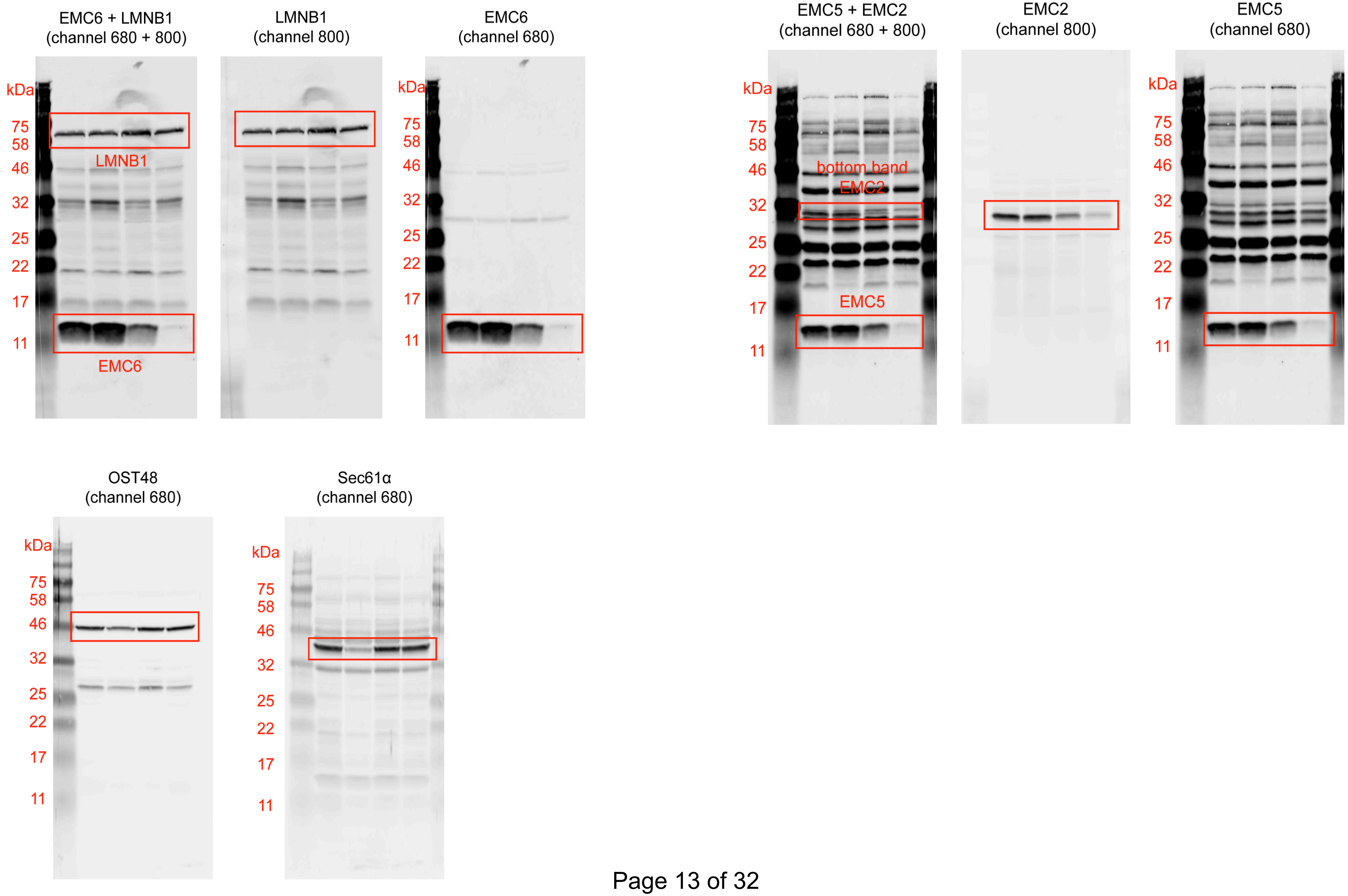

Microsomes

OST48 + EMC2
(channel 680 + 800)

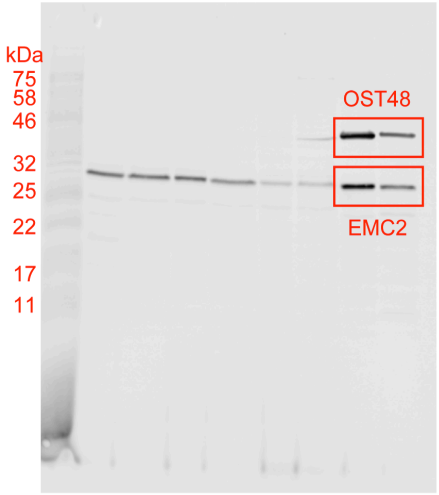

EMC2
(channel 800)

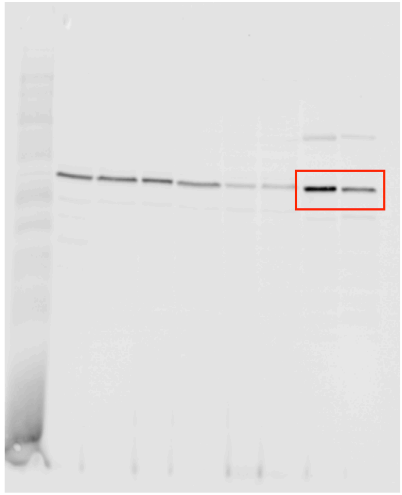

OST48
(channel 680)

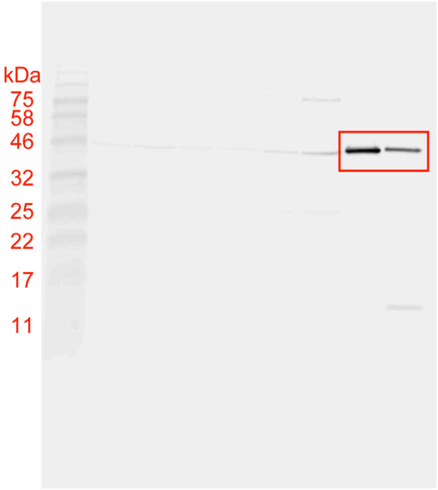

Sec61α
(channel 680)

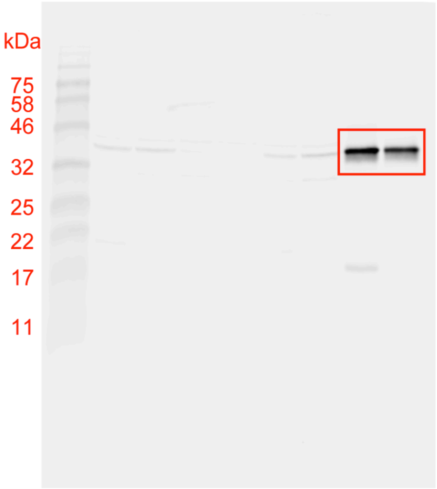

EMC6 + LMNB1
(channel 680 + 800)

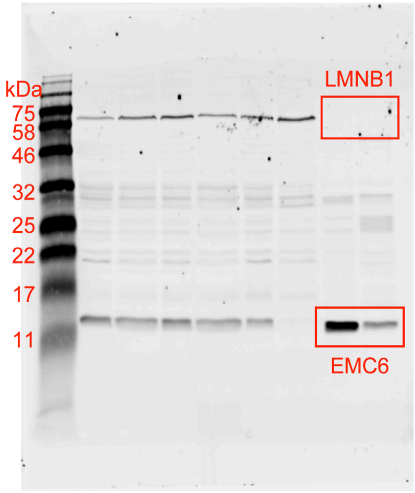

LMNB1
(channel 800)

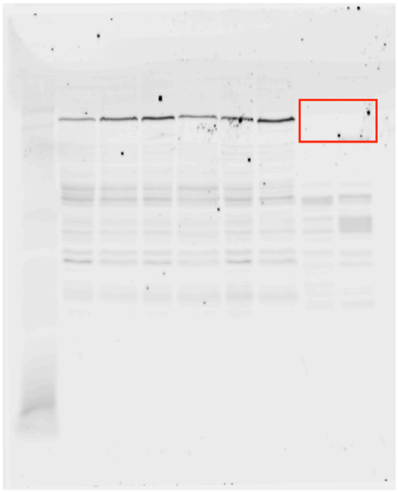

EMC6
(channel 680)

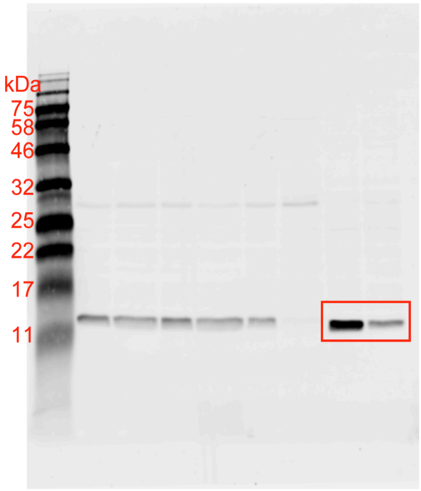

EMC5
(channel 680)

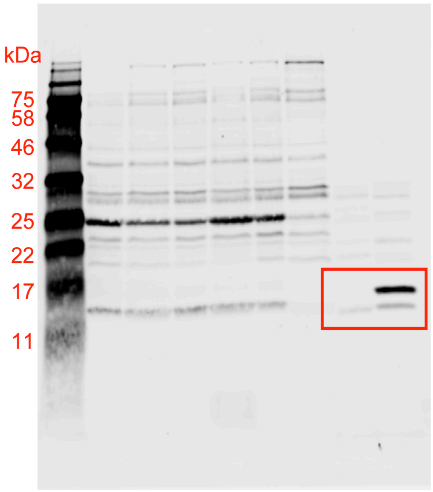

Fig. 3c: Phosphorimages of NT, Se61α-kd, EMC2-kd, EMC5-kd (+/- Ipom-F) insertion assay

ASGR1OPG2

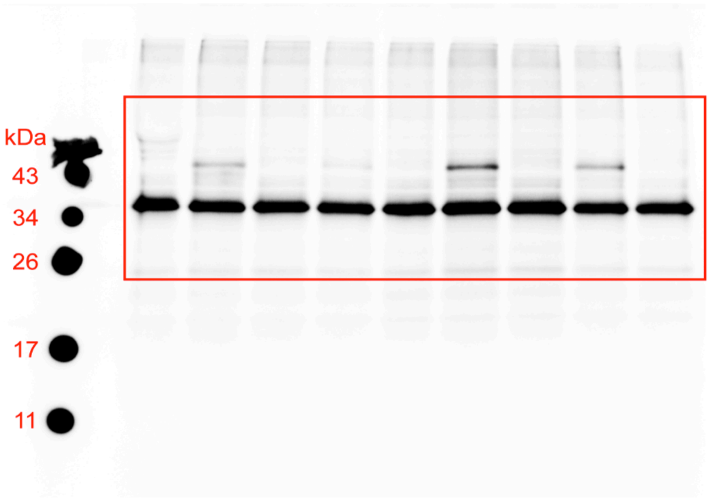

Cytb5OPG2

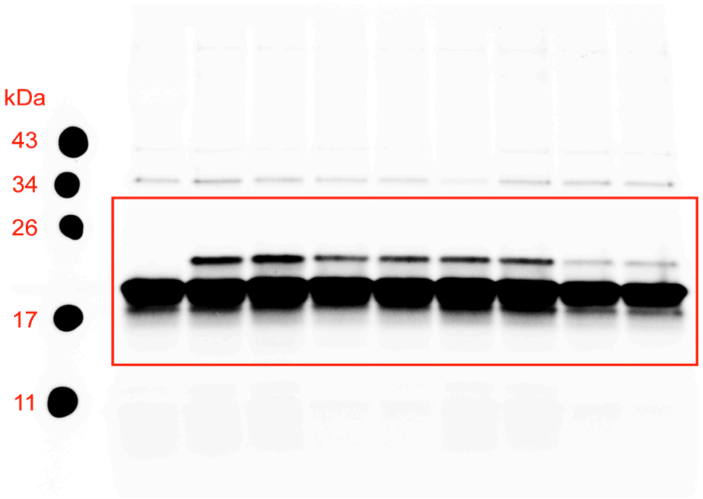

Fig. 4b: Phosphorimages of NT, Se61α-kd, EMC2-kd, EMC5-kd (+/- Ipom-F) insertion assay

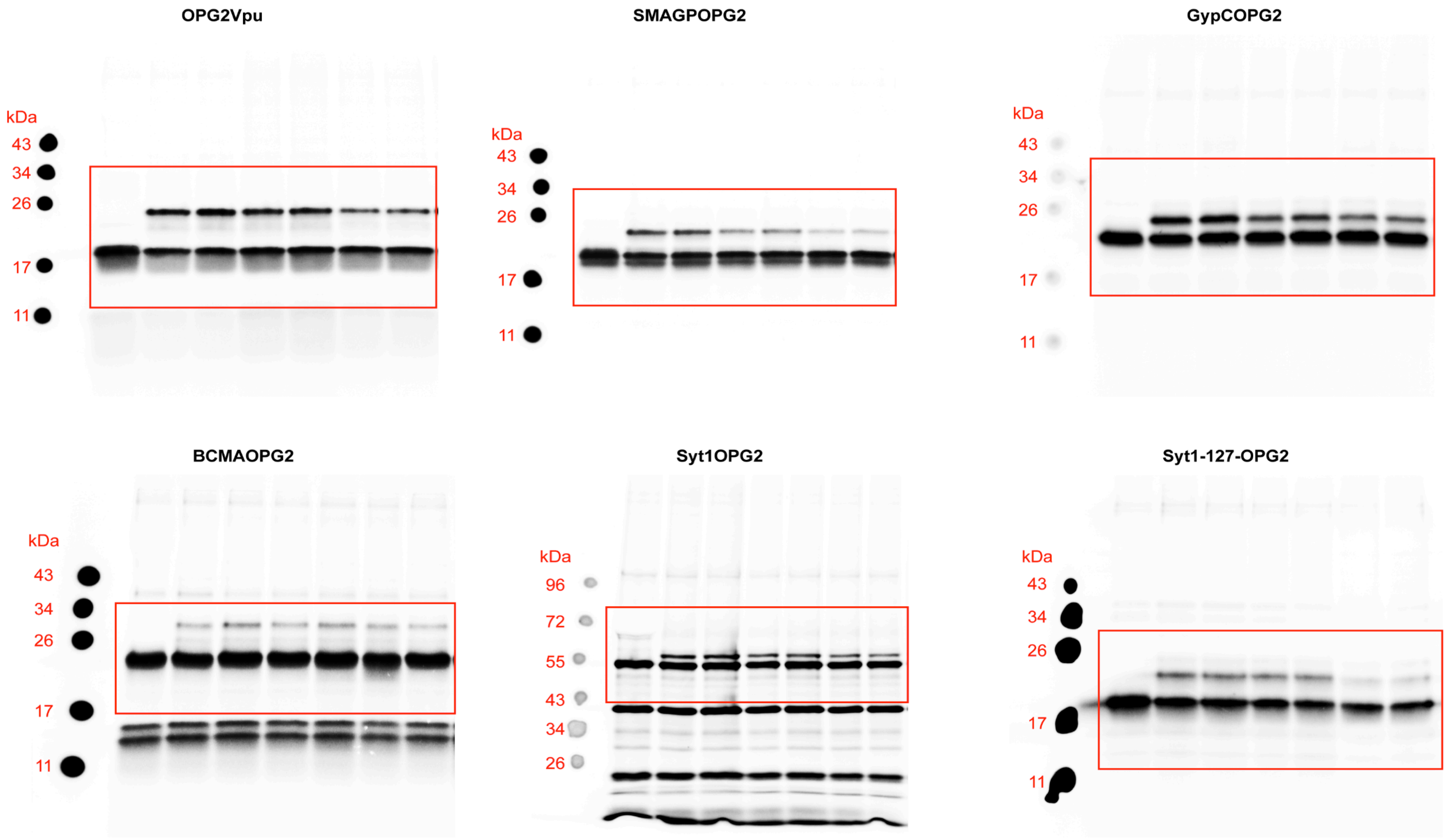

Fig. 5a: Efficiency of double knockdowns; NT, Se61α+EMC2-kd, Se61α+EMC5

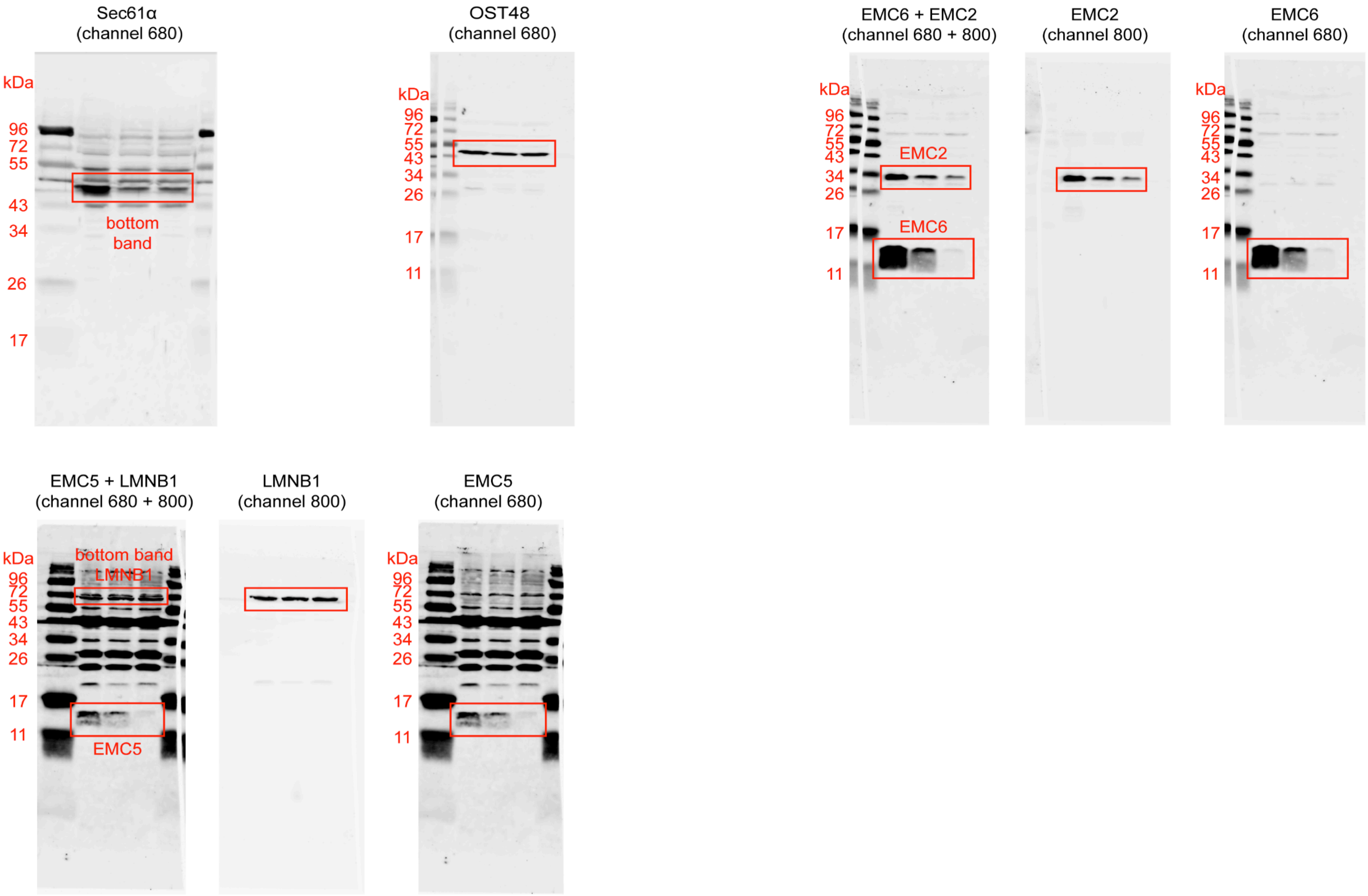

Fig. 5c: Phosphorimages of NT, Se61α+EMC2-kd, Se61α+EMC5-kd (+/- Ipom-F) insertion assay

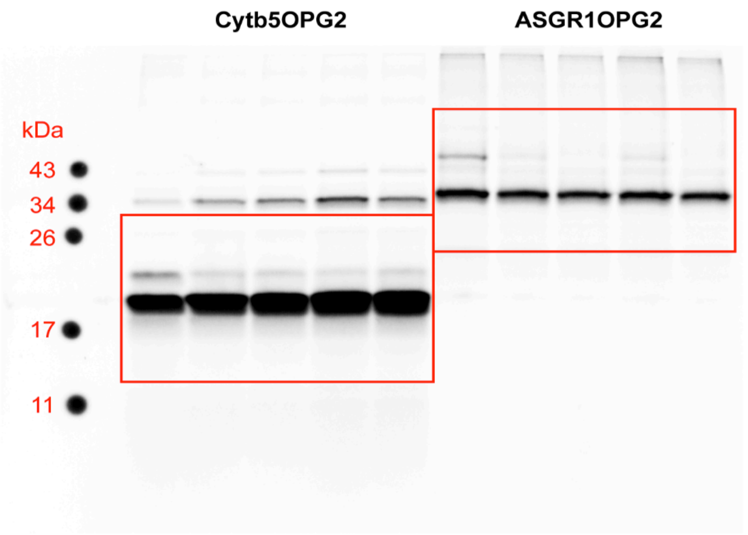

Fig. 5e: Phosphorimages of NT, Se61α+EMC2-kd, Se61α+EMC5-kd (+/- Ipom-F) insertion assay

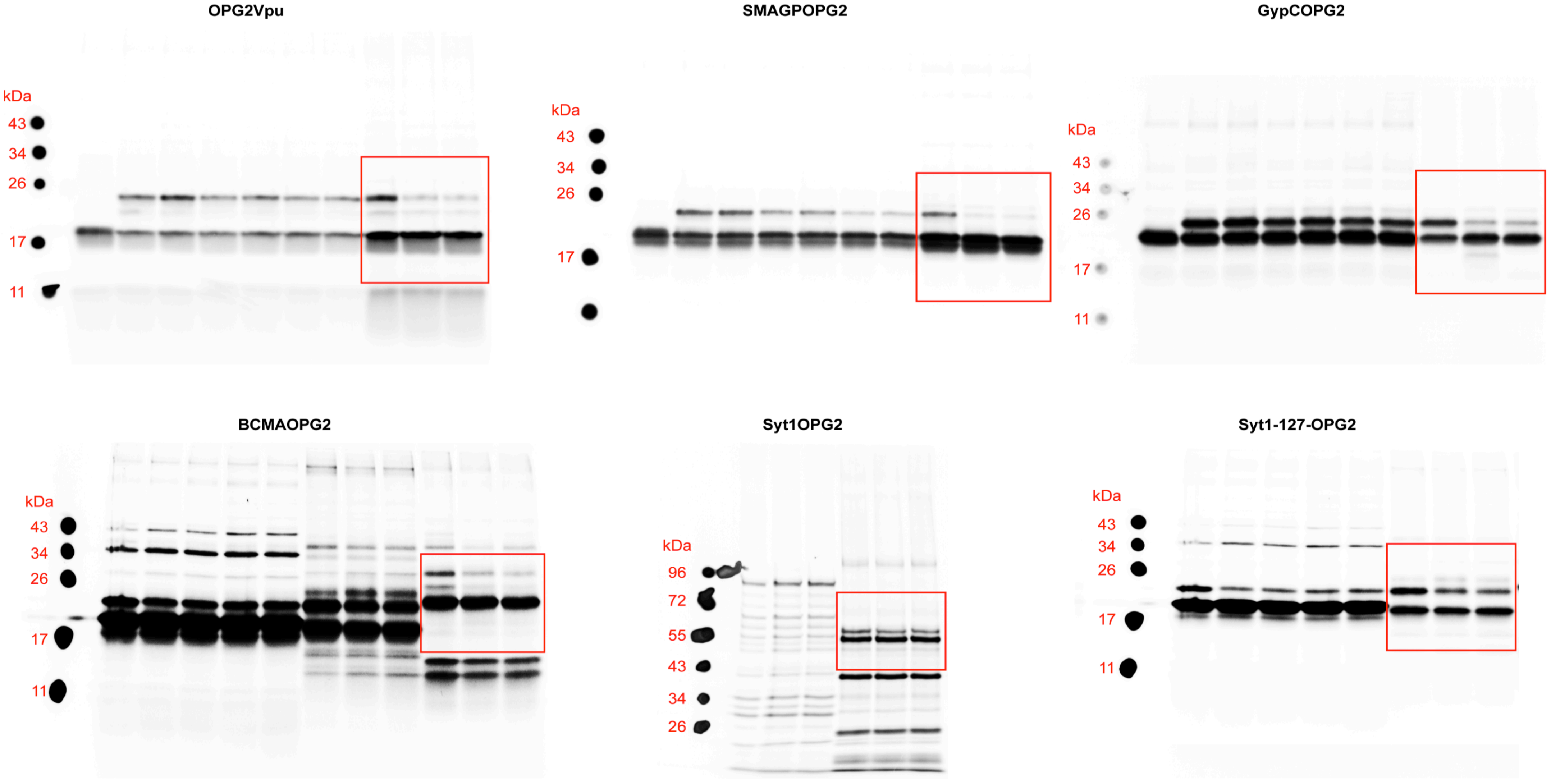

Fig. 6b: Efficiency of single and combined SRα knockdowns; NT, SRα-kd, SRα+EMC5-kd, SRα+Sec61α-kd

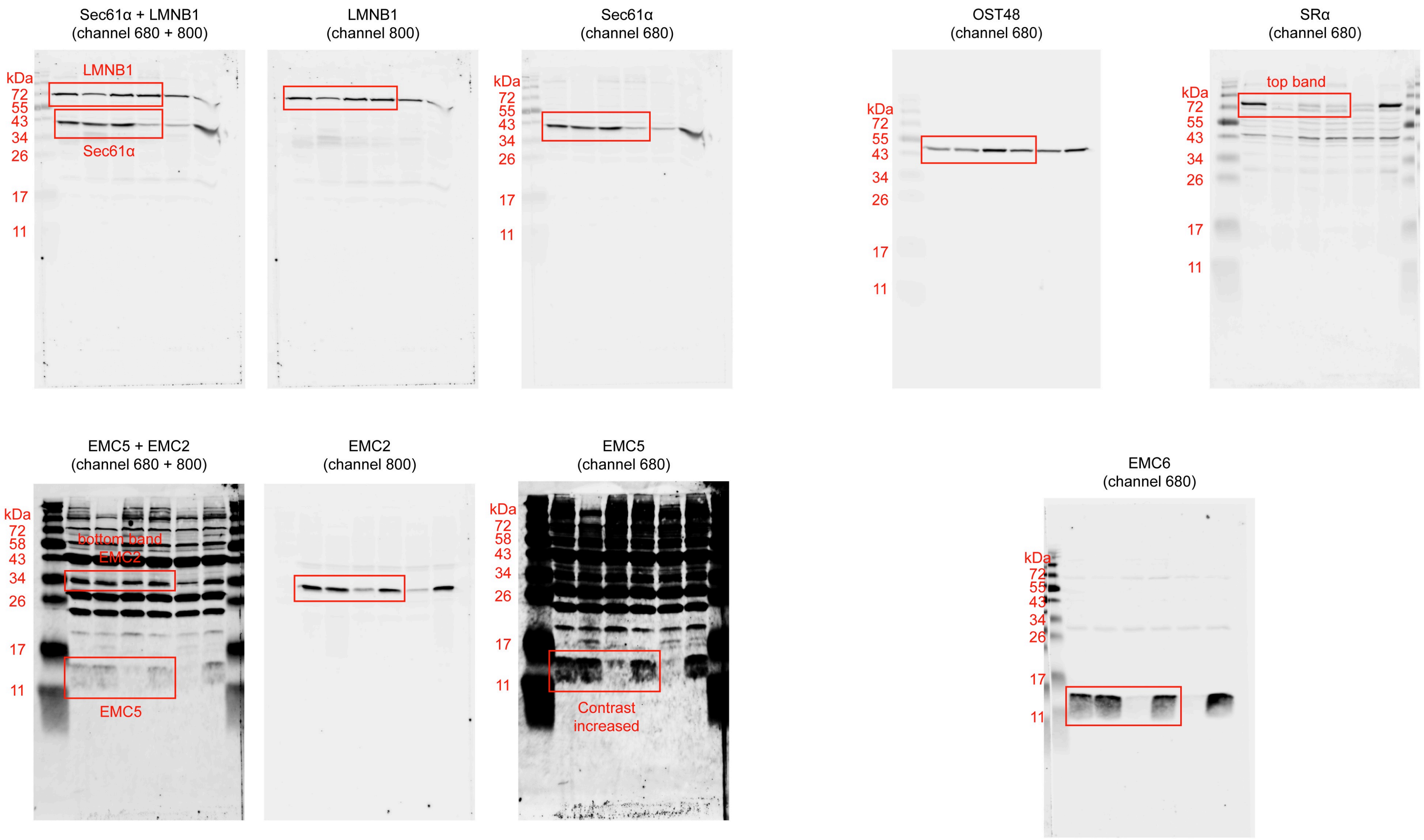

Fig. 6d: Phosphorimages of NT, SRα-kd, SRα+EMC5-kd, SRα+Sec61α-kd insertion assay

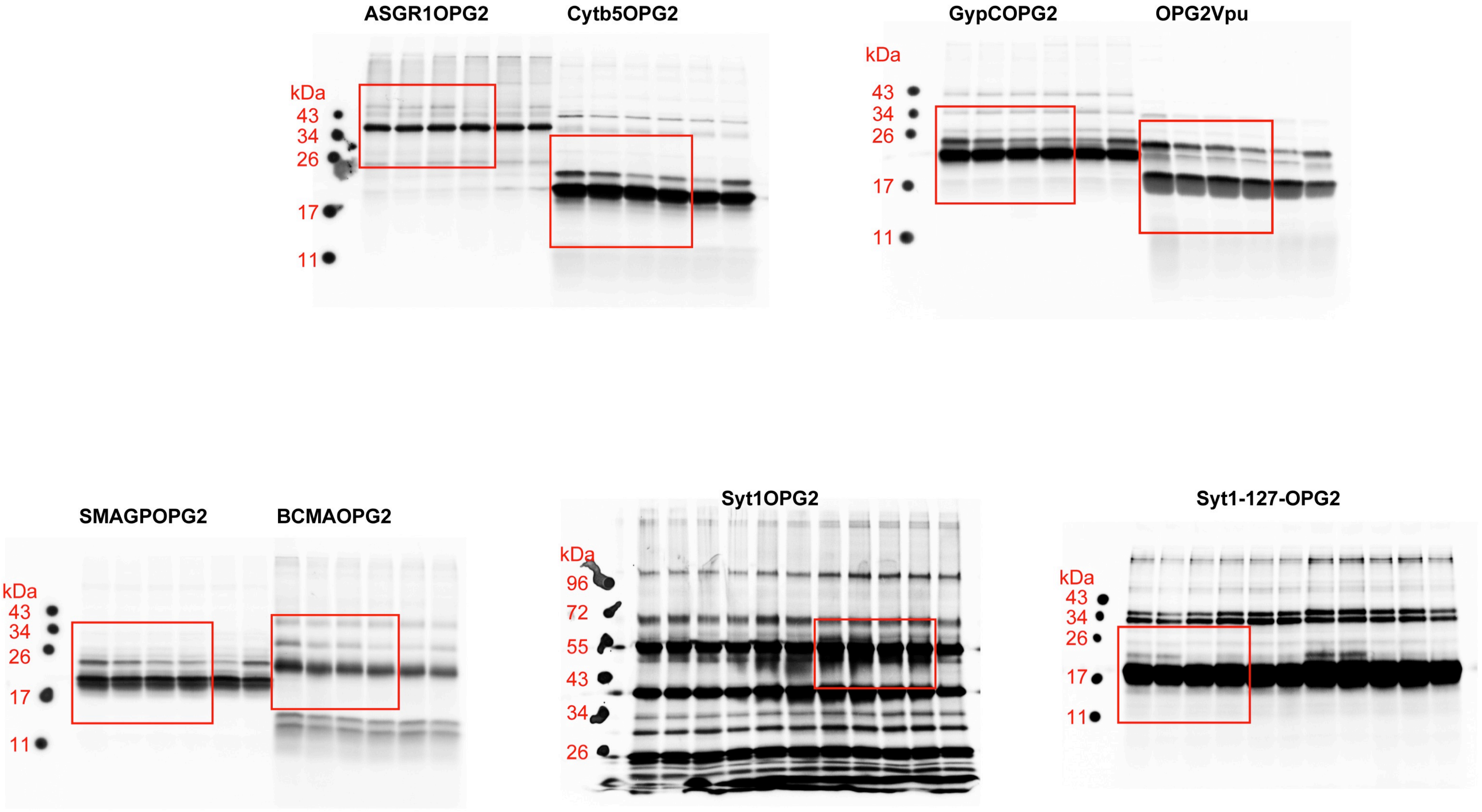

Supplementary Fig. 1: Phosphorimages of +/- Ipom-F insertion assay in microsomes and SP cells

Microsomes

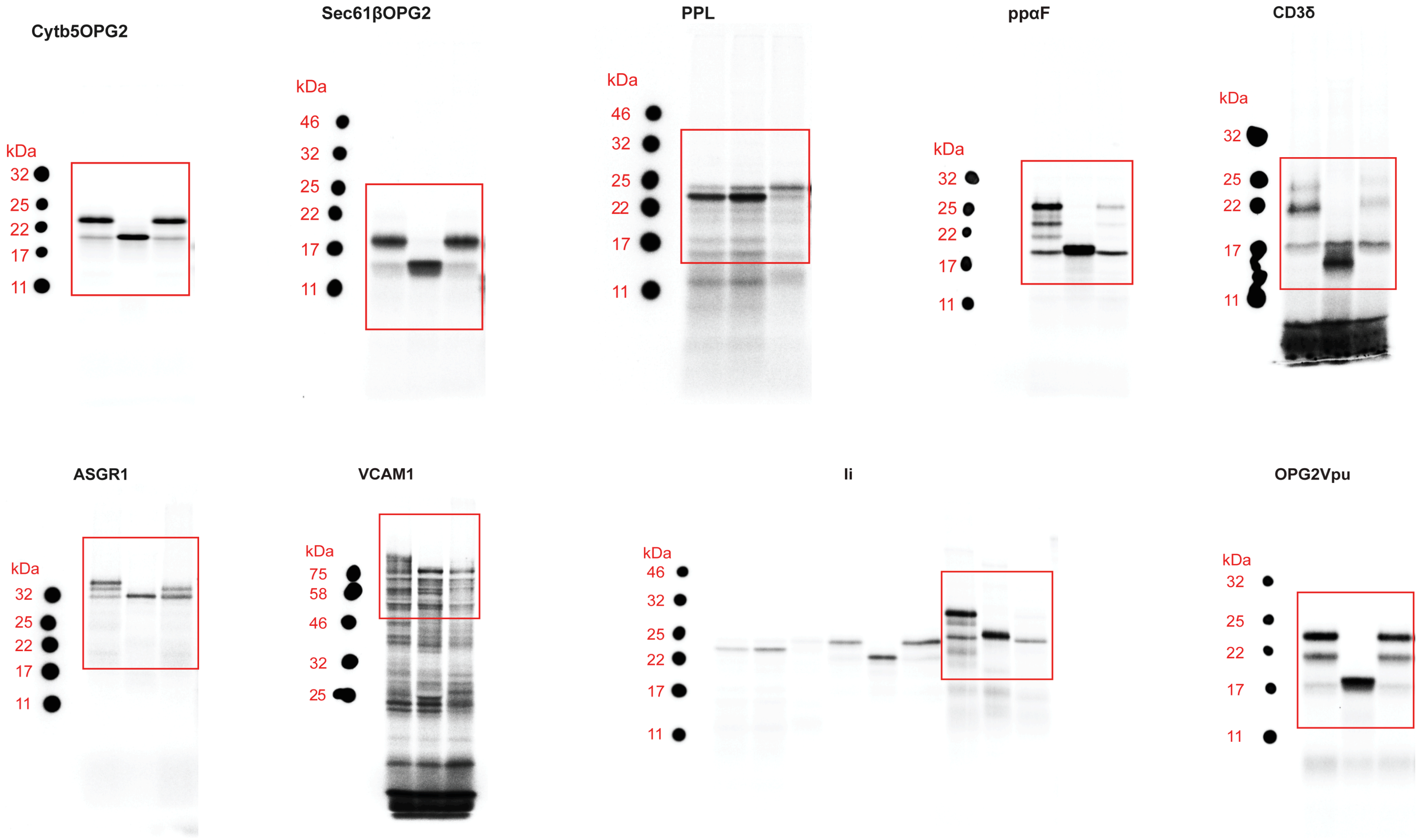

SP Cells

VCAM1

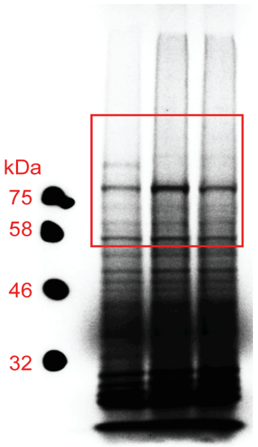

Ii

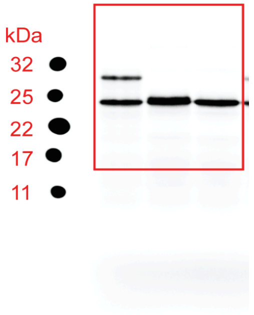

OPG2Vpu

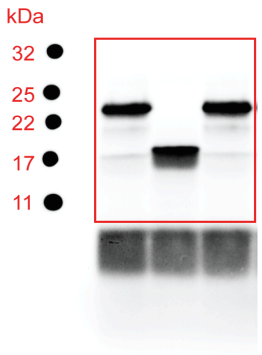

Supplementary Fig. 3b: Phosphorimages of +/- Ipom-F insertion assay in microsomes

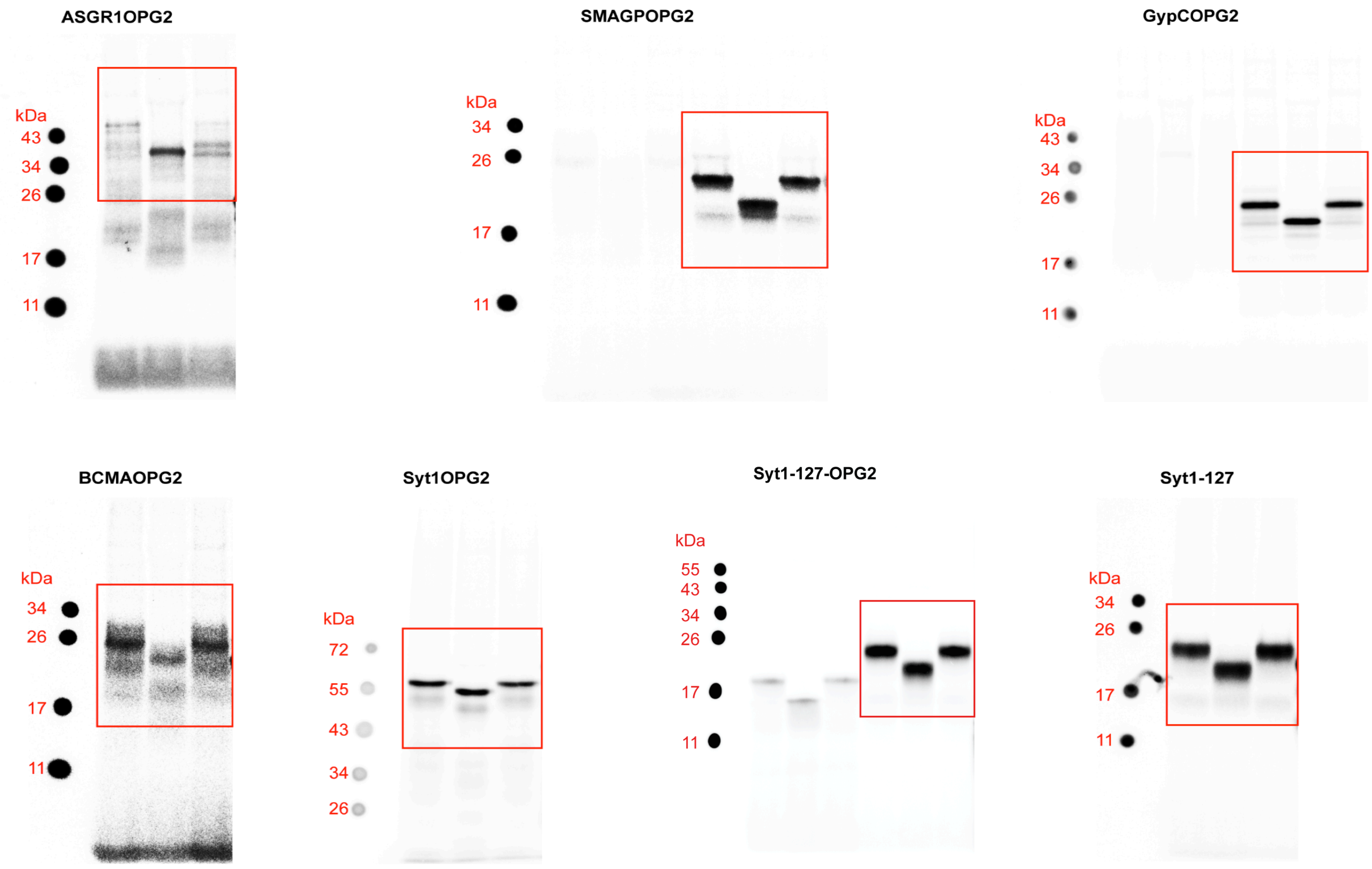

Supplementary Fig. 3c: Phosphorimages to determine the immunoprecipitation efficiency OPG2-tagged TMPs

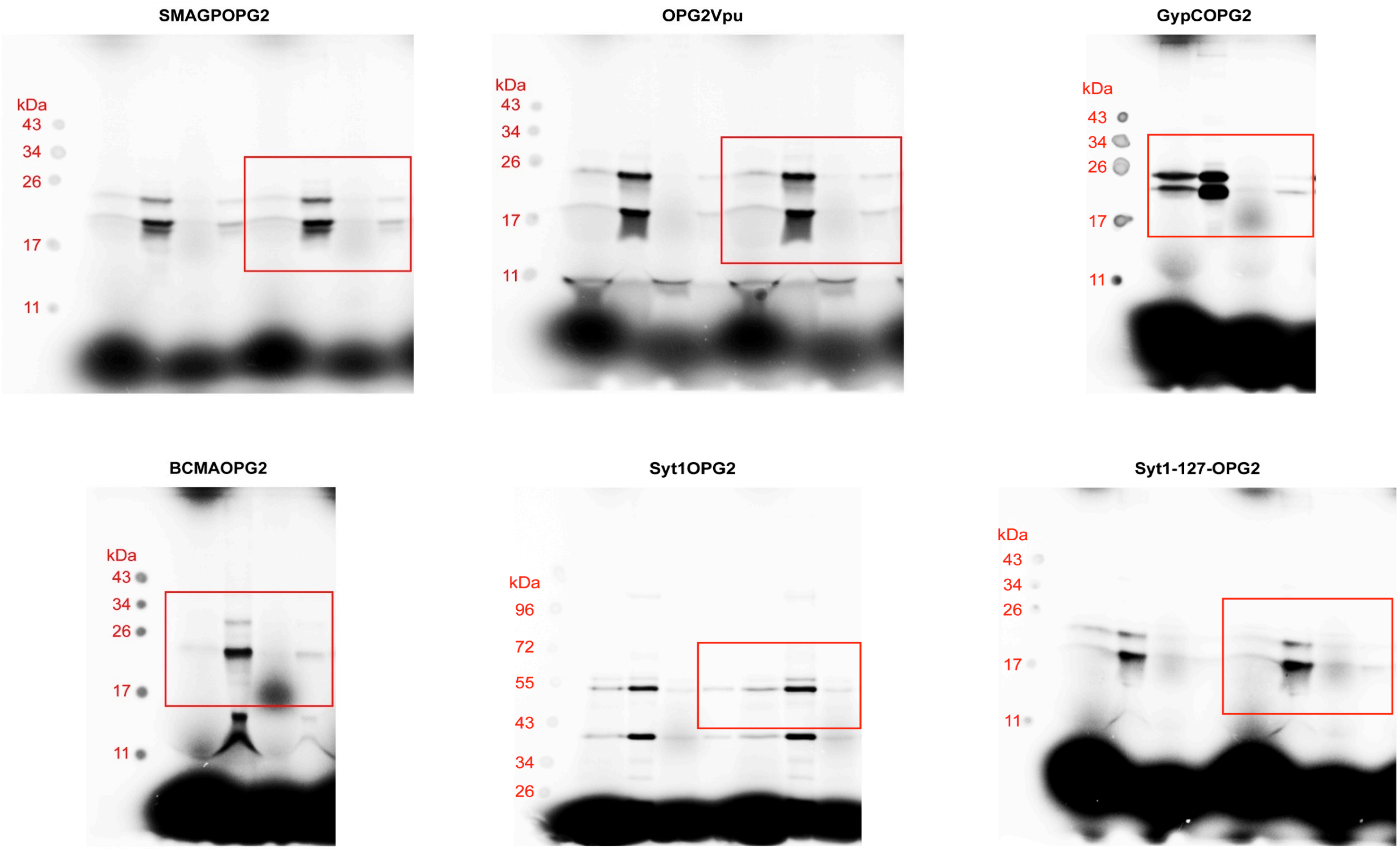

Supplementary Fig. 5b: NT, Sec61α-kd, EMC2-kd, EMC5-kd, Sec61α+EMC2-kd, Sec61α+EMC5-kd, SRα-kd, SRα+EMC5-kd, SRα+Sec61α-kd Western blots

Single knockdowns of Sec61α, EMC2 and EMC5 (lanes 1-4, left panels)

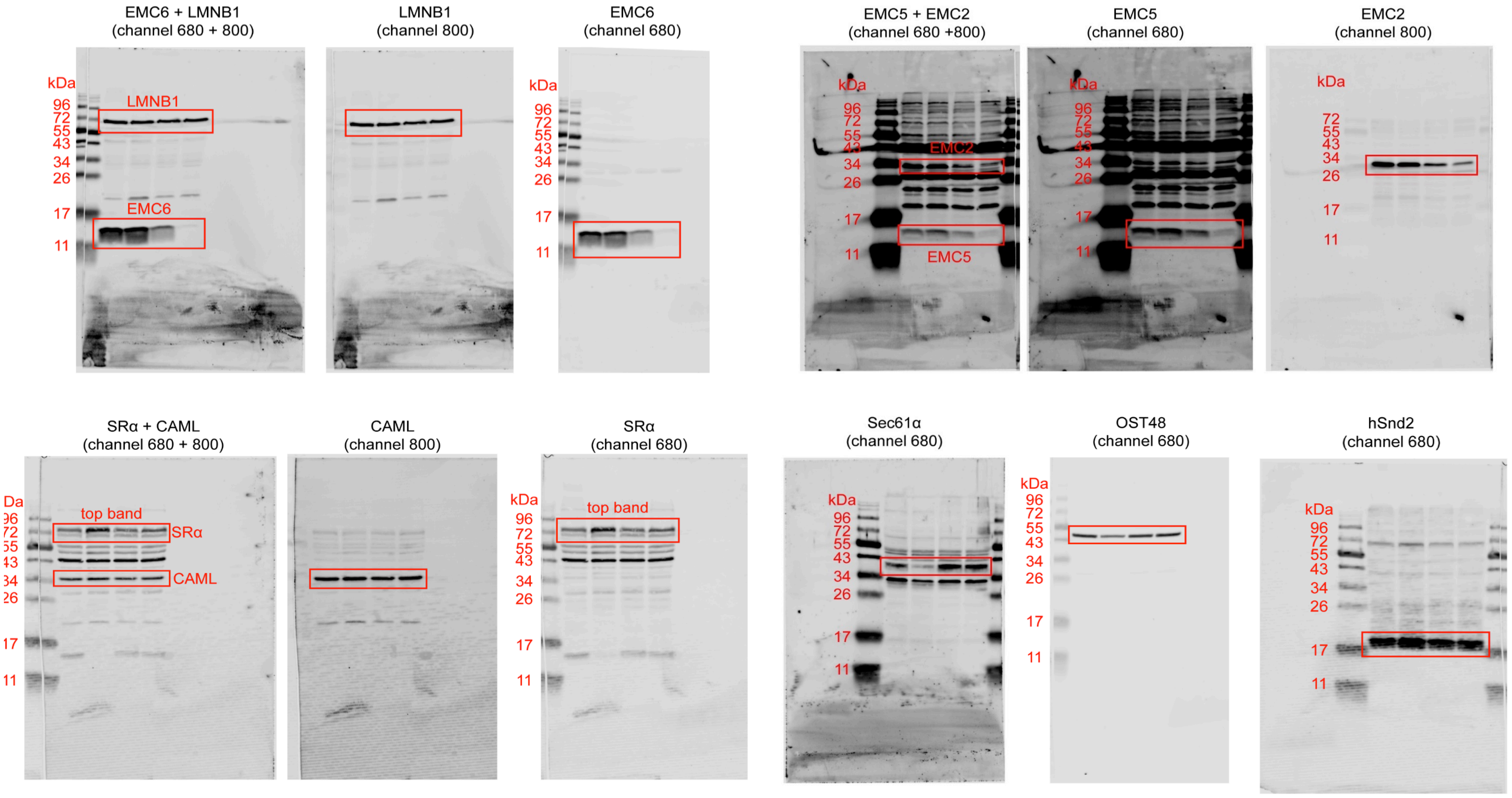

Double knockdowns of Sec61α+EMC2 and Sec61α+EMC5 (lanes 5-7, left panels)

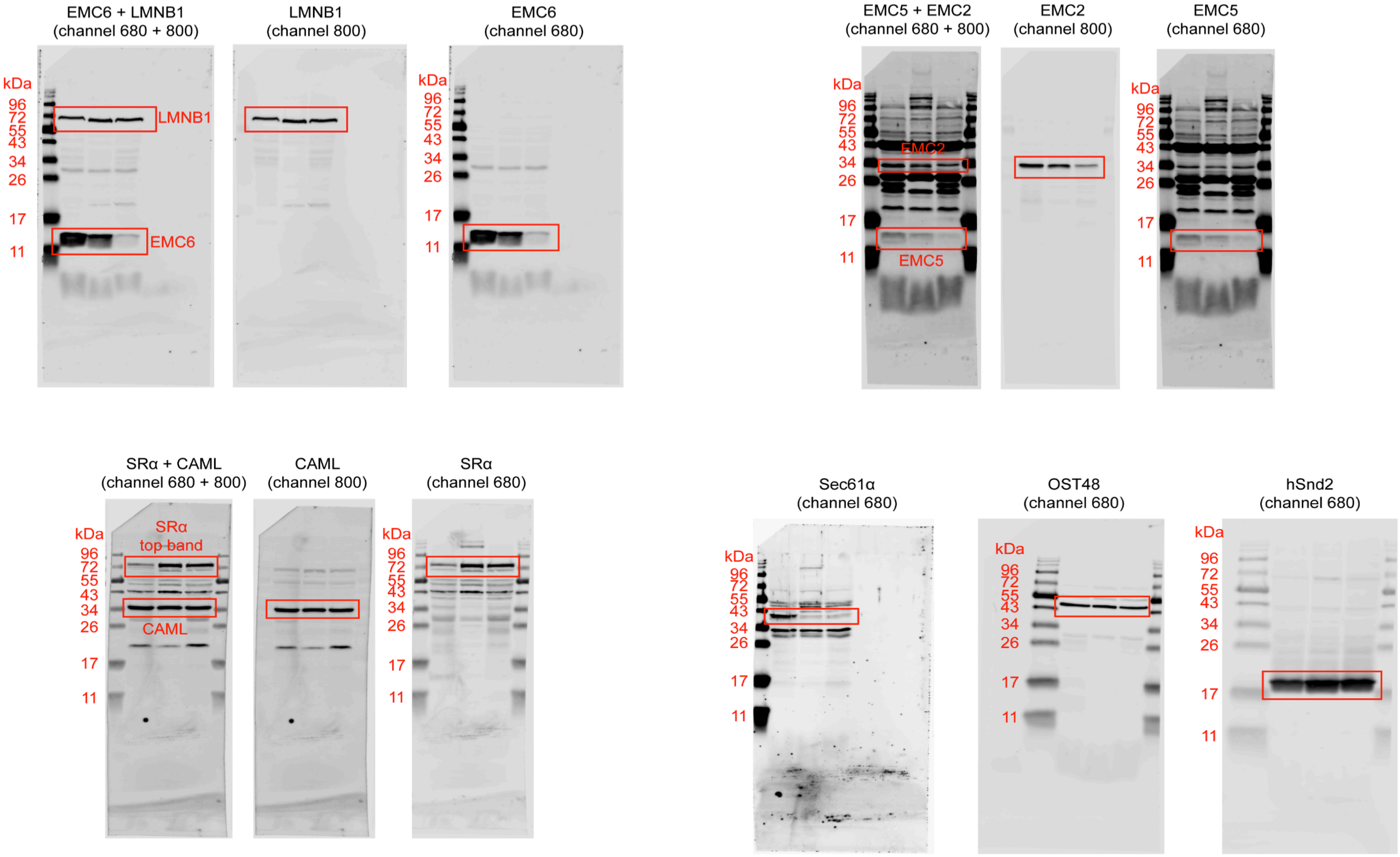

Knockdowns of SRα (lanes 8-9, left panels)

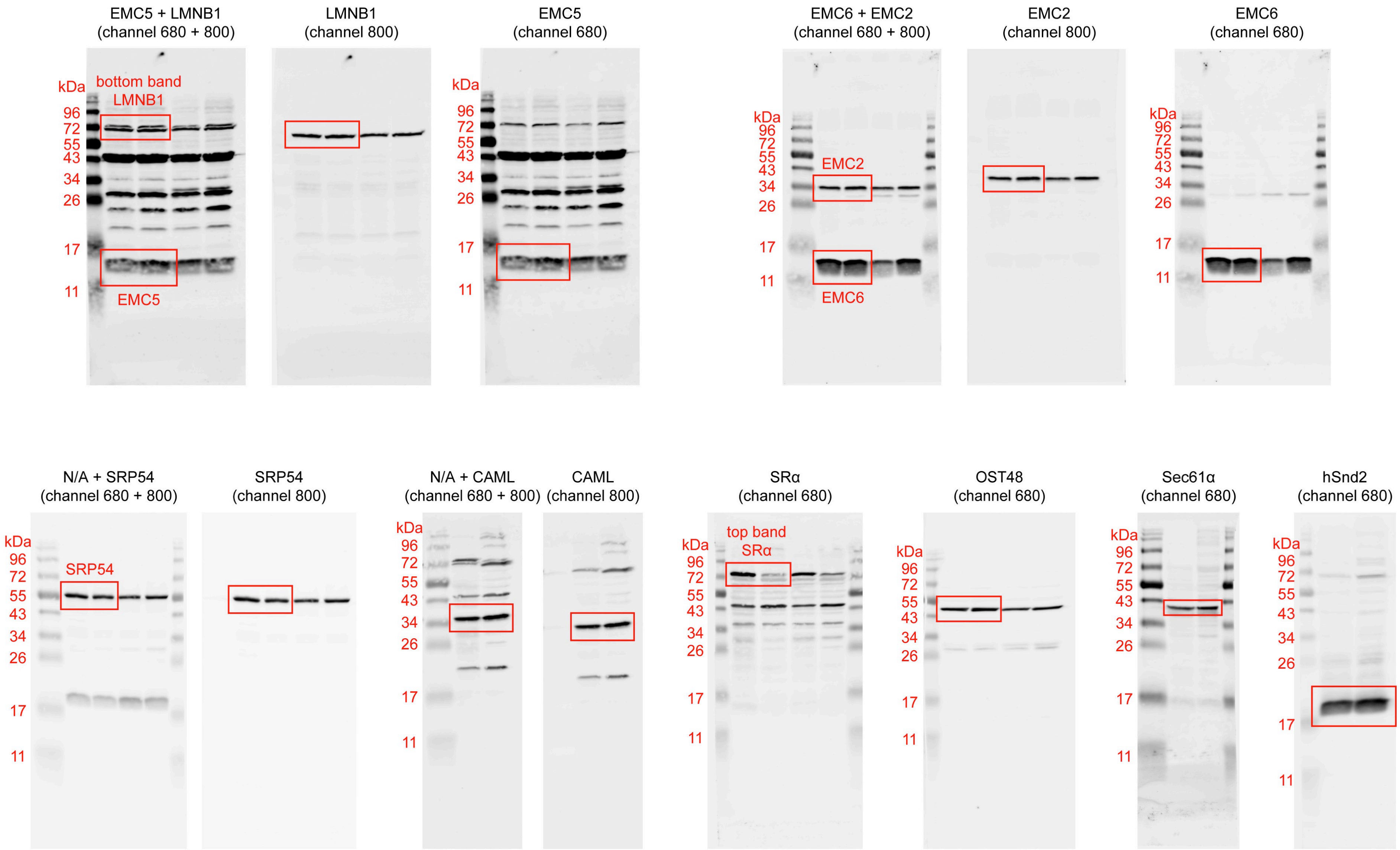

Knockdowns of SRα+EMC5, SRα+Sec61α (lanes 10-12, left panels)

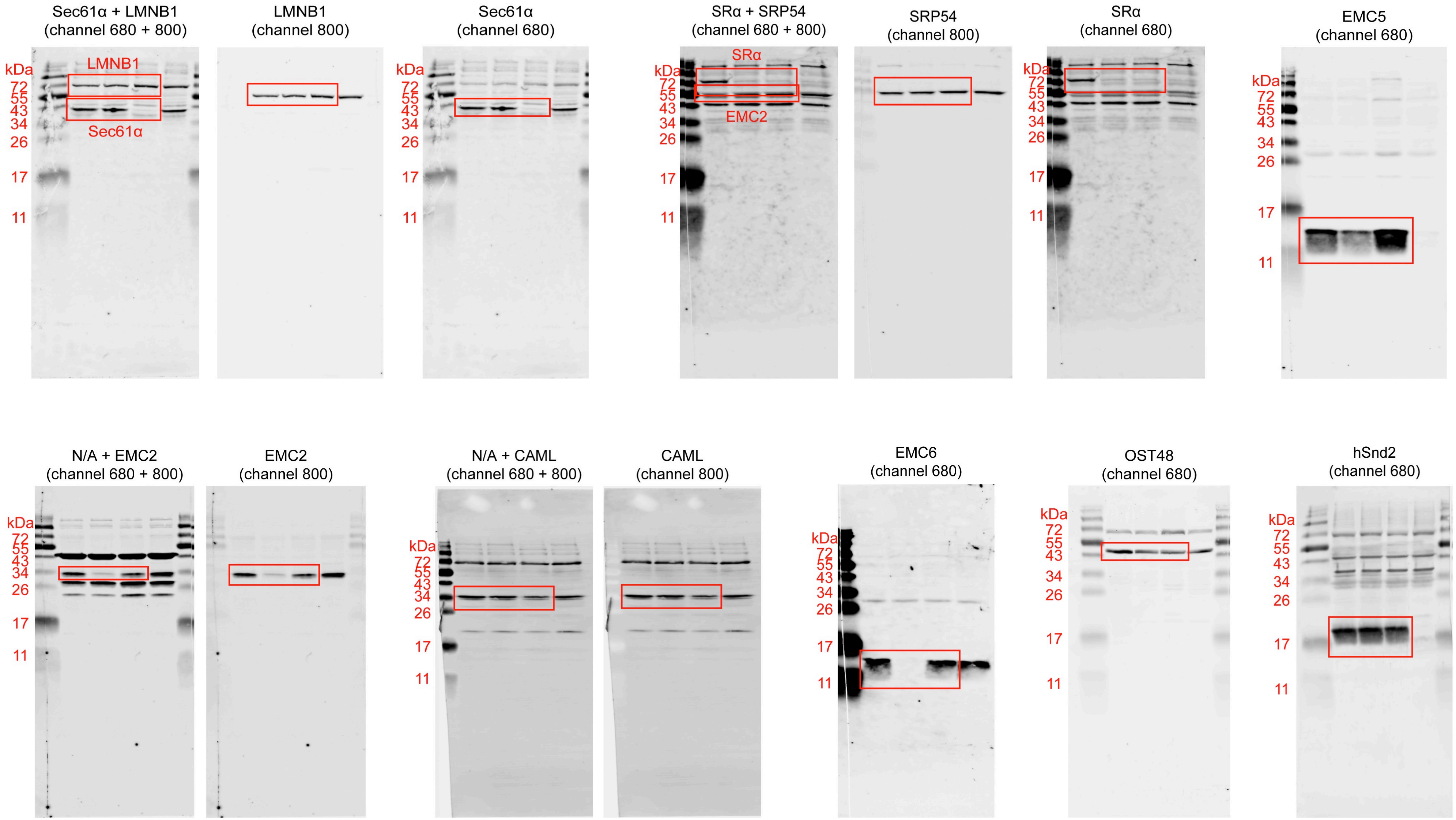

Knockdowns of Sec61α, EMC2, EMC5, Sec61α+EMC5 (lanes 1-4, 7, right panels)

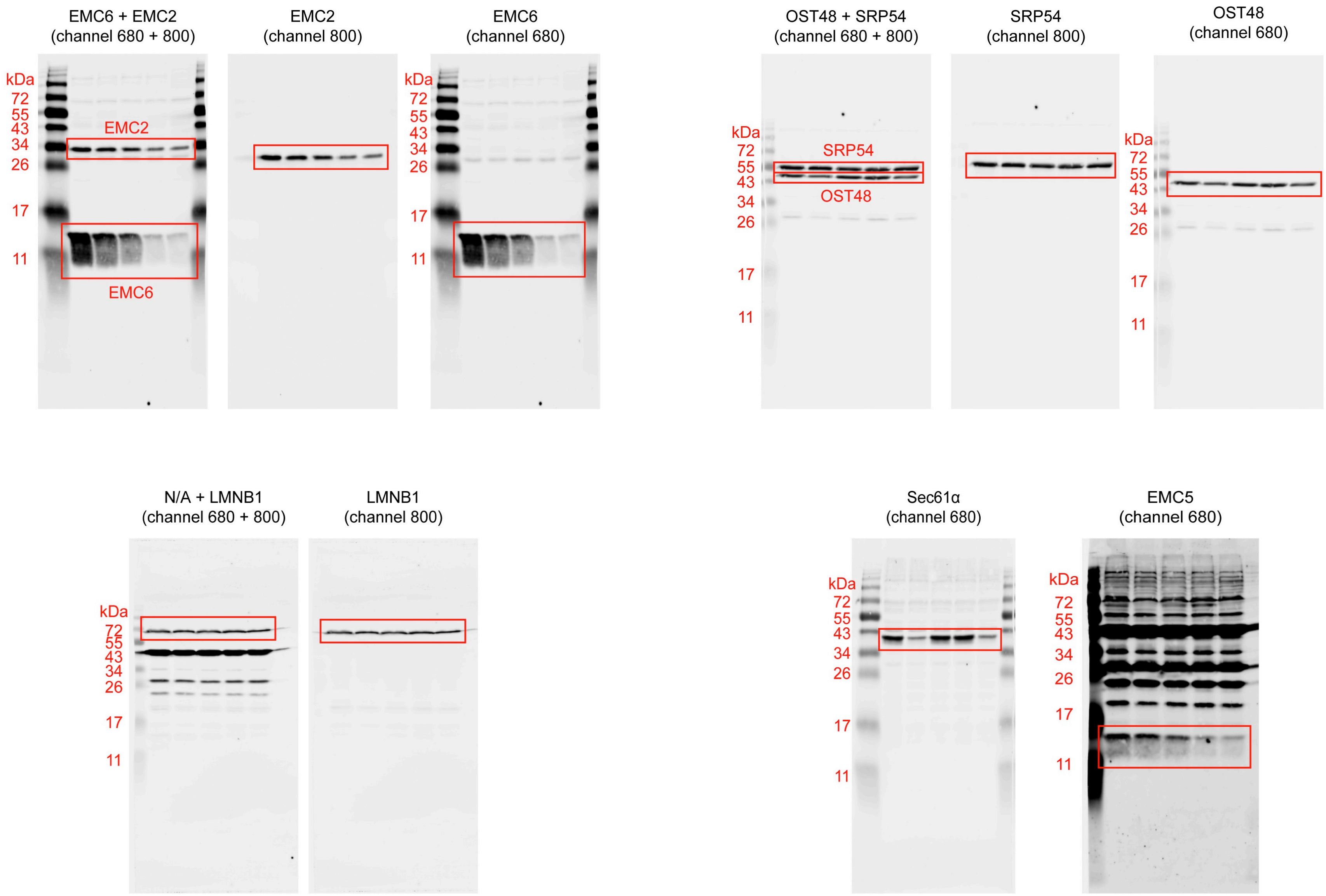

Supplementary Figure 6

Uncropped and unedited blot/gel images

Fig. 1d: Phosphorimages of +/- Ipom-F insertion assay in microsomes

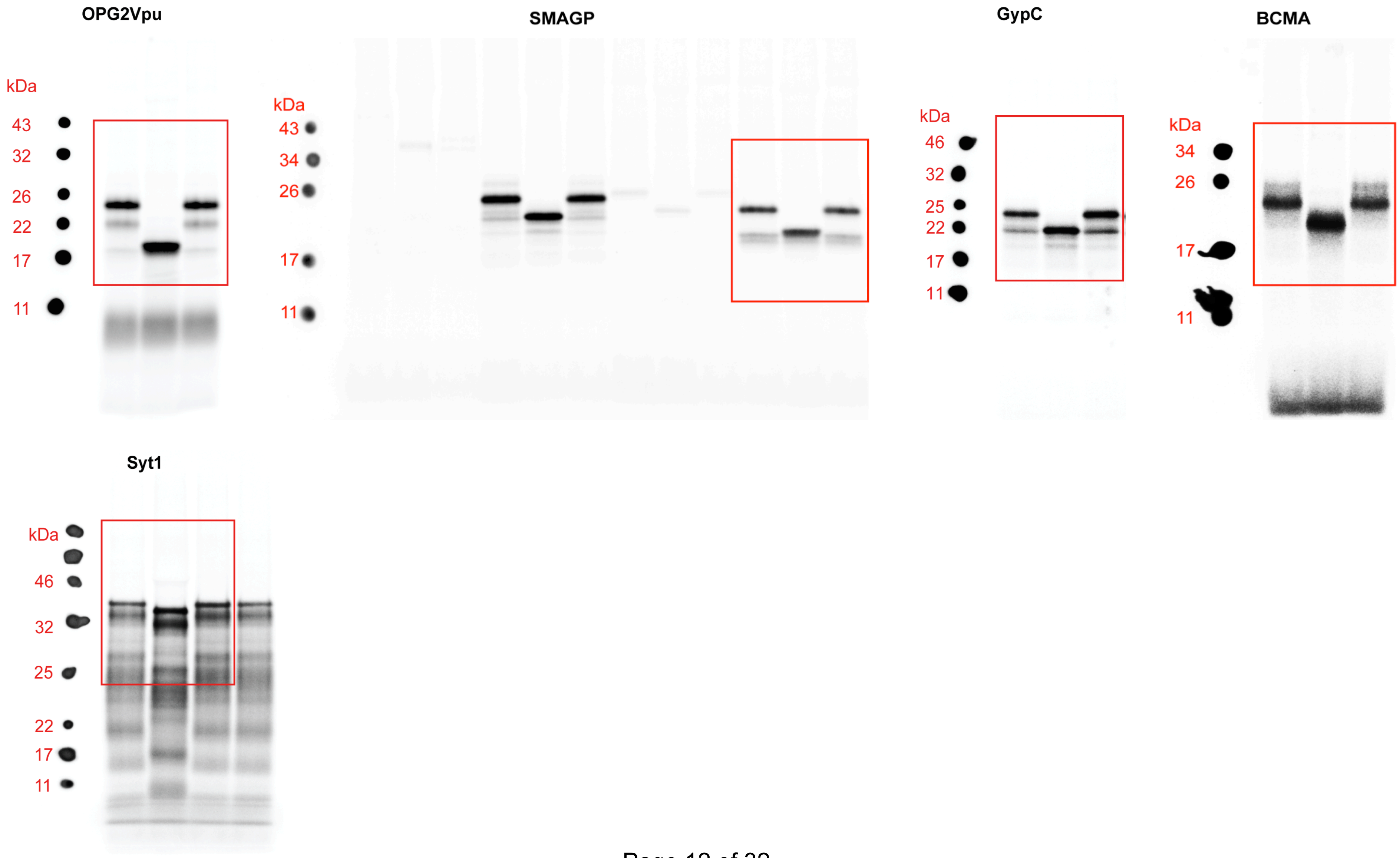

Fig. 2c: Efficiency of single knockdowns; NT, Se61α-kd, EMC2-kd, EMC5-kd

<u>SP Cells</u>

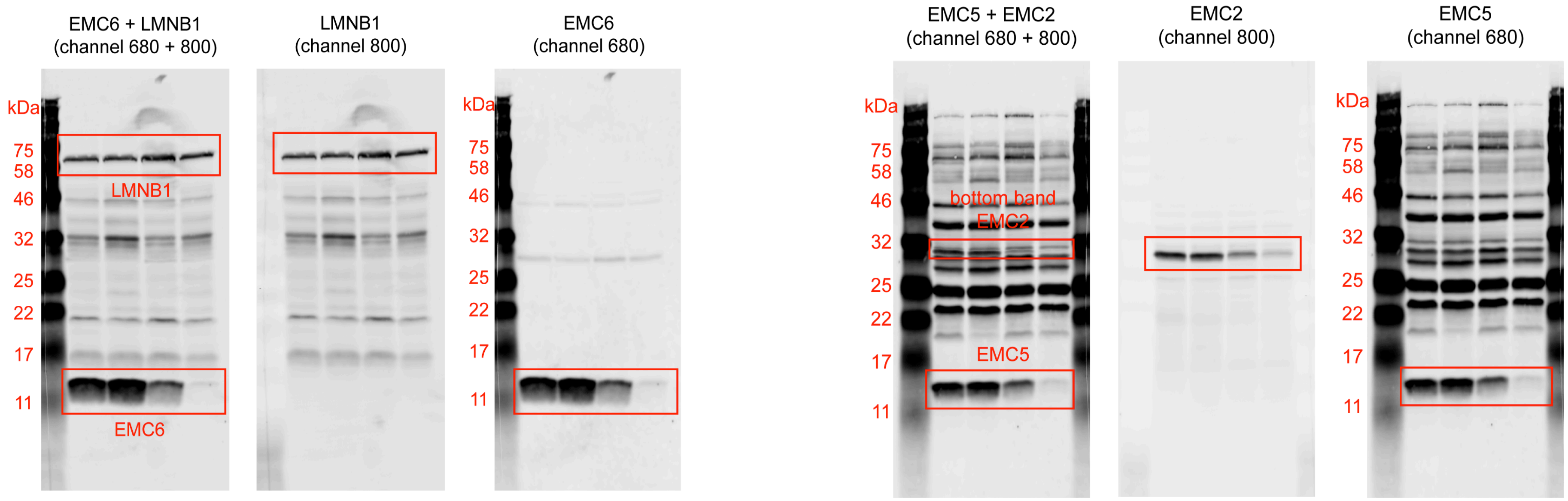

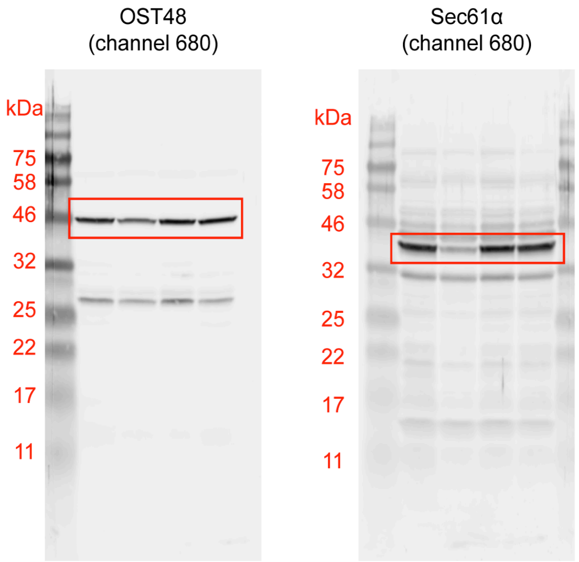

Microsomes

OST48 + EMC2
(channel 680 + 800)

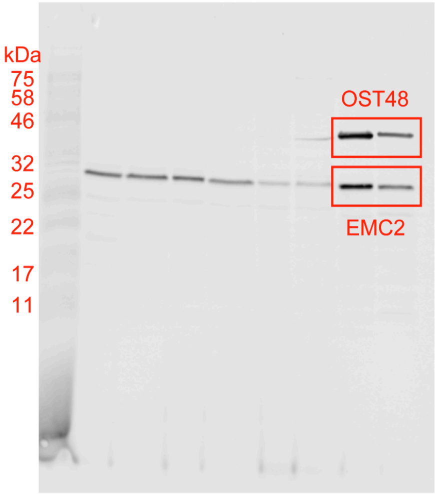

EMC2
(channel 800)

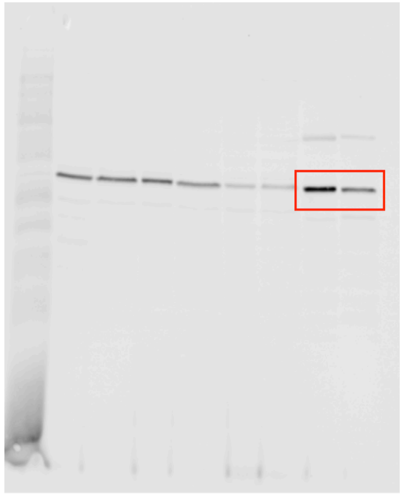

OST48
(channel 680)

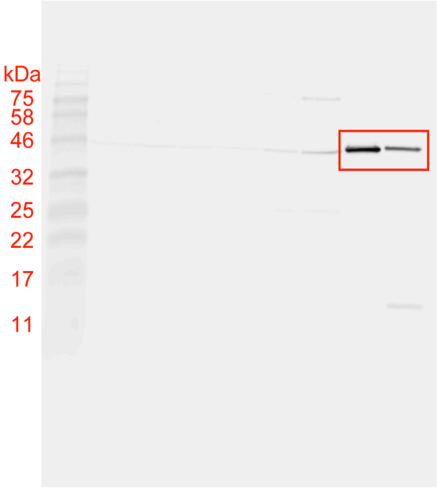

Sec61α
(channel 680)

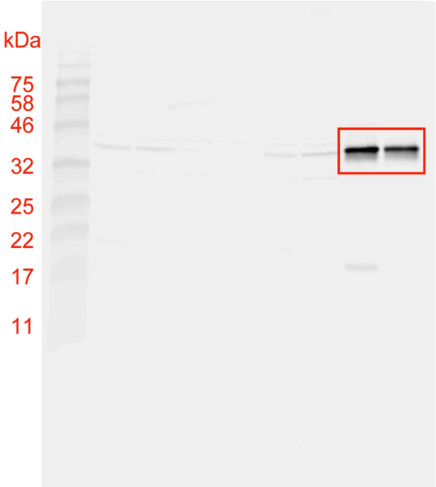

EMC6 + LMNB1
(channel 680 + 800)

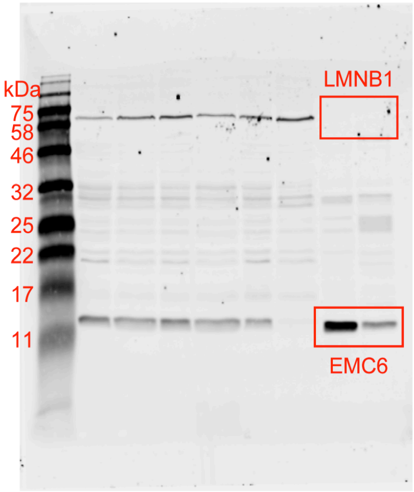

LMNB1
(channel 800)

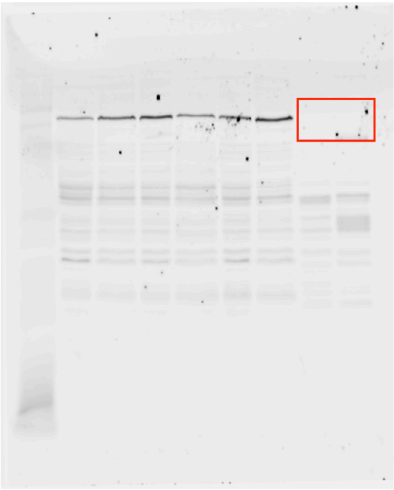

EMC6
(channel 680)

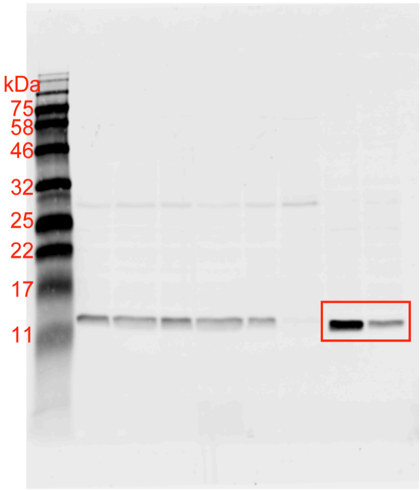

EMC5
(channel 680)

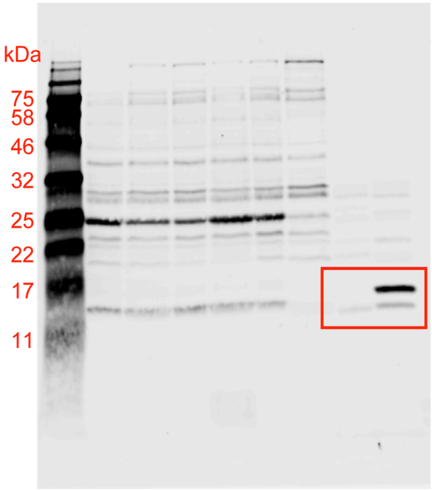

Fig. 3c: Phosphorimages of NT, Se61α-kd, EMC2-kd, EMC5-kd (+/- Ipom-F) insertion assay

ASGR1OPG2

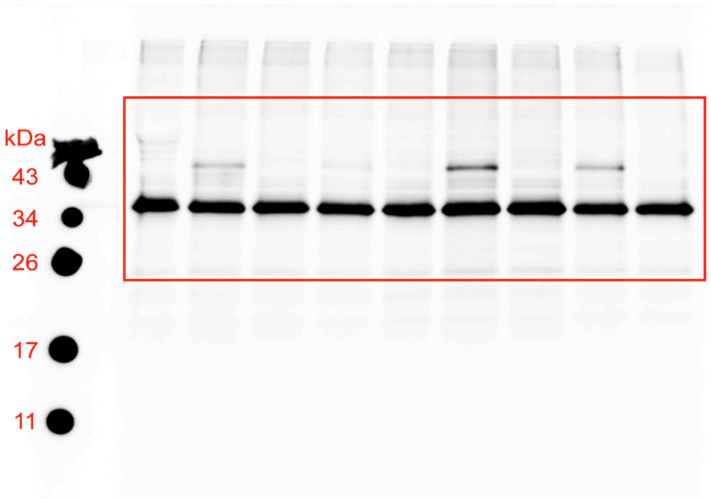

Cytb5OPG2

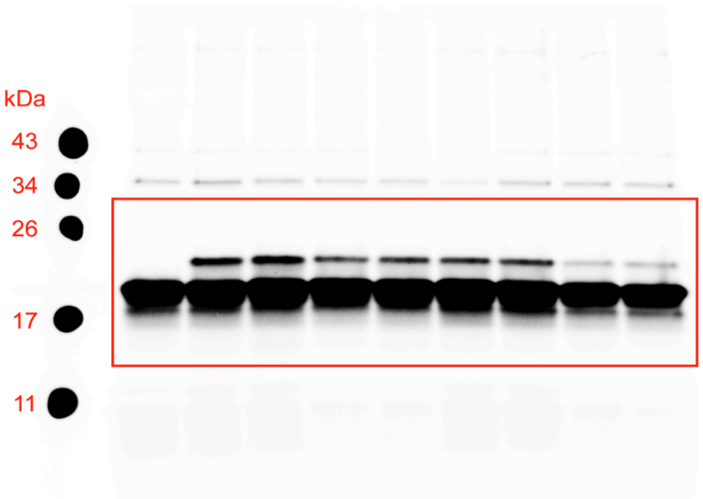

Fig. 4b: Phosphorimages of NT, Se61α-kd, EMC2-kd, EMC5-kd (+/- Ipom-F) insertion assay

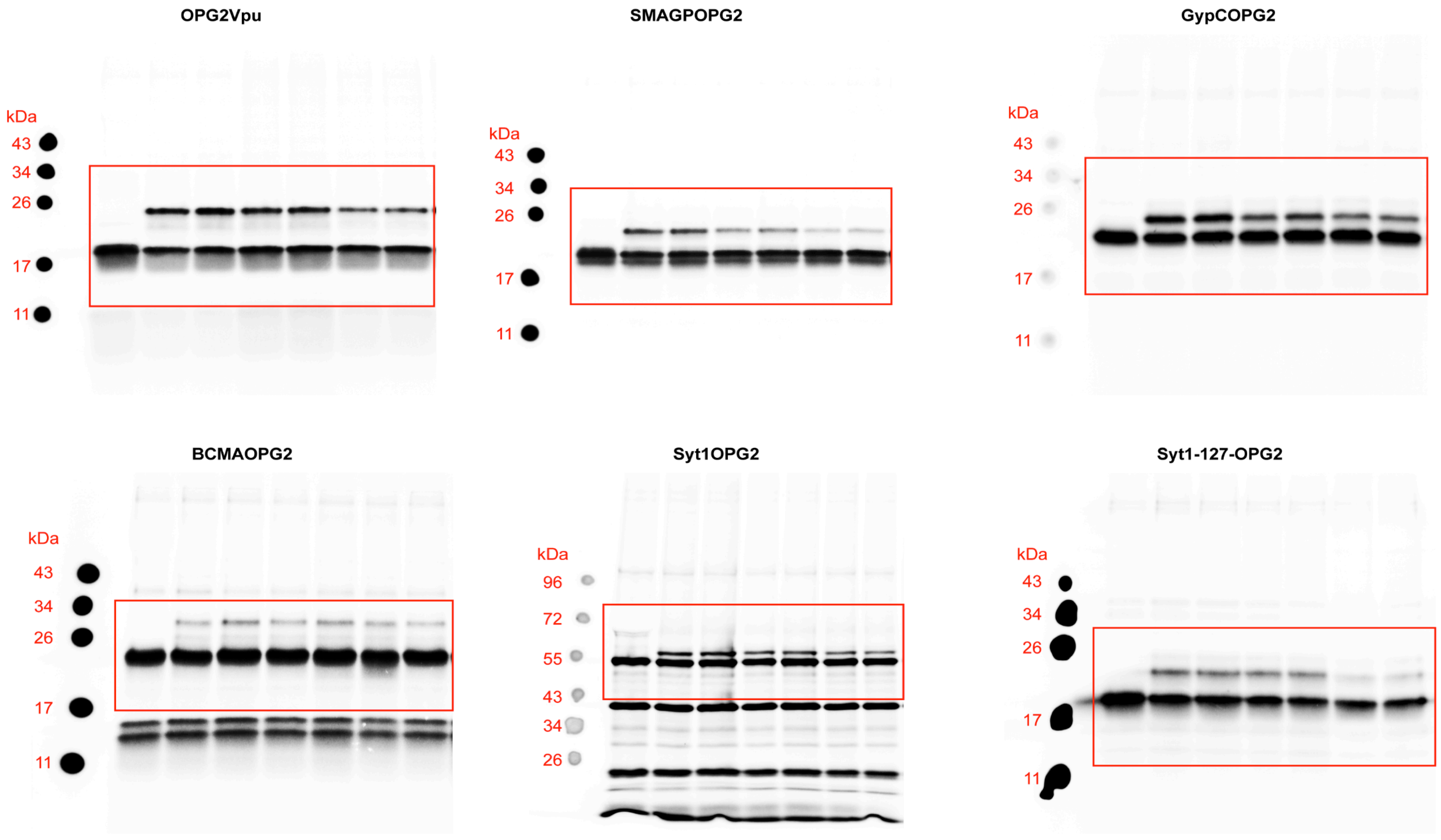

Fig. 5a: Efficiency of double knockdowns; NT, Se61α+EMC2-kd, Se61α+EMC5

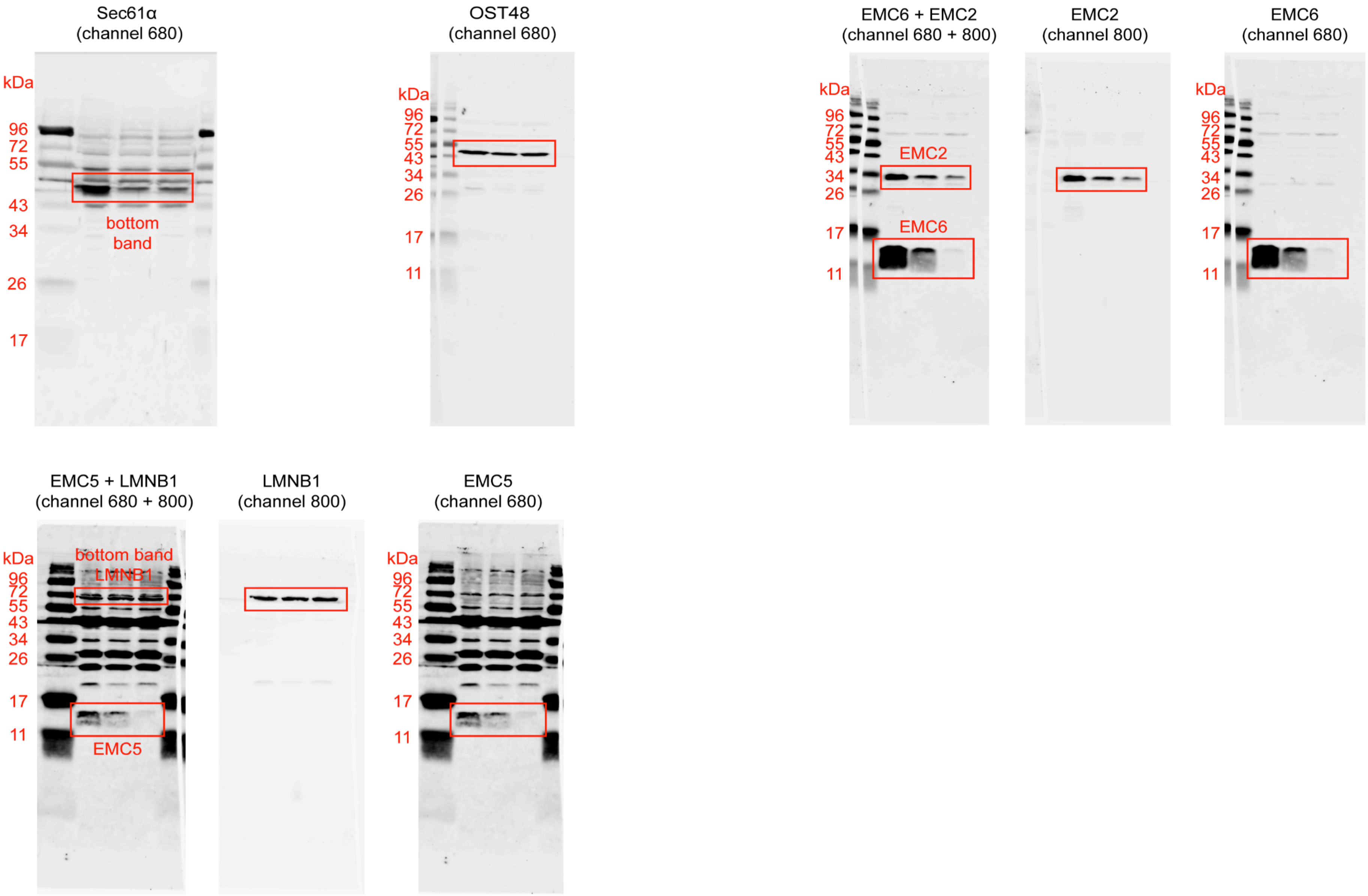

Fig. 5c: Phosphorimages of NT, Se61α+EMC2-kd, Se61α+EMC5-kd (+/- Ipom-F) insertion assay

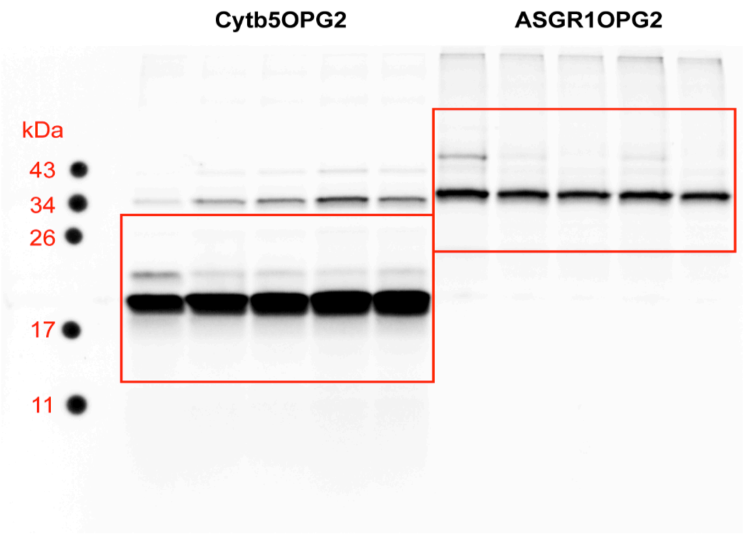

Fig. 5e: Phosphorimages of NT, Se61α+EMC2-kd, Se61α+EMC5-kd (+/- Ipom-F) insertion assay

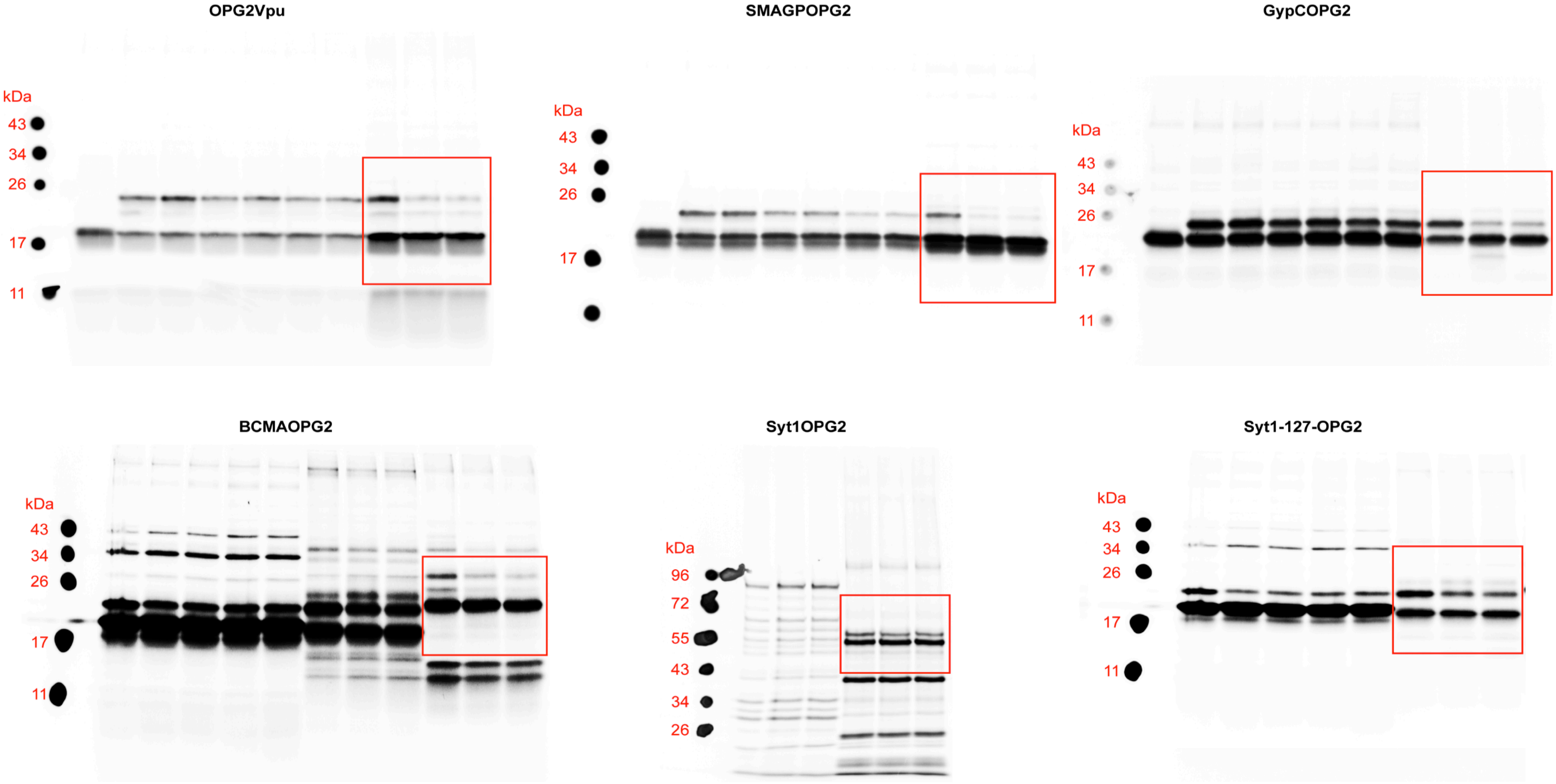

Fig. 6b: Efficiency of single and combined SRα knockdowns; NT, SRα-kd, SRα+EMC5-kd, SRα+Sec61α-kd

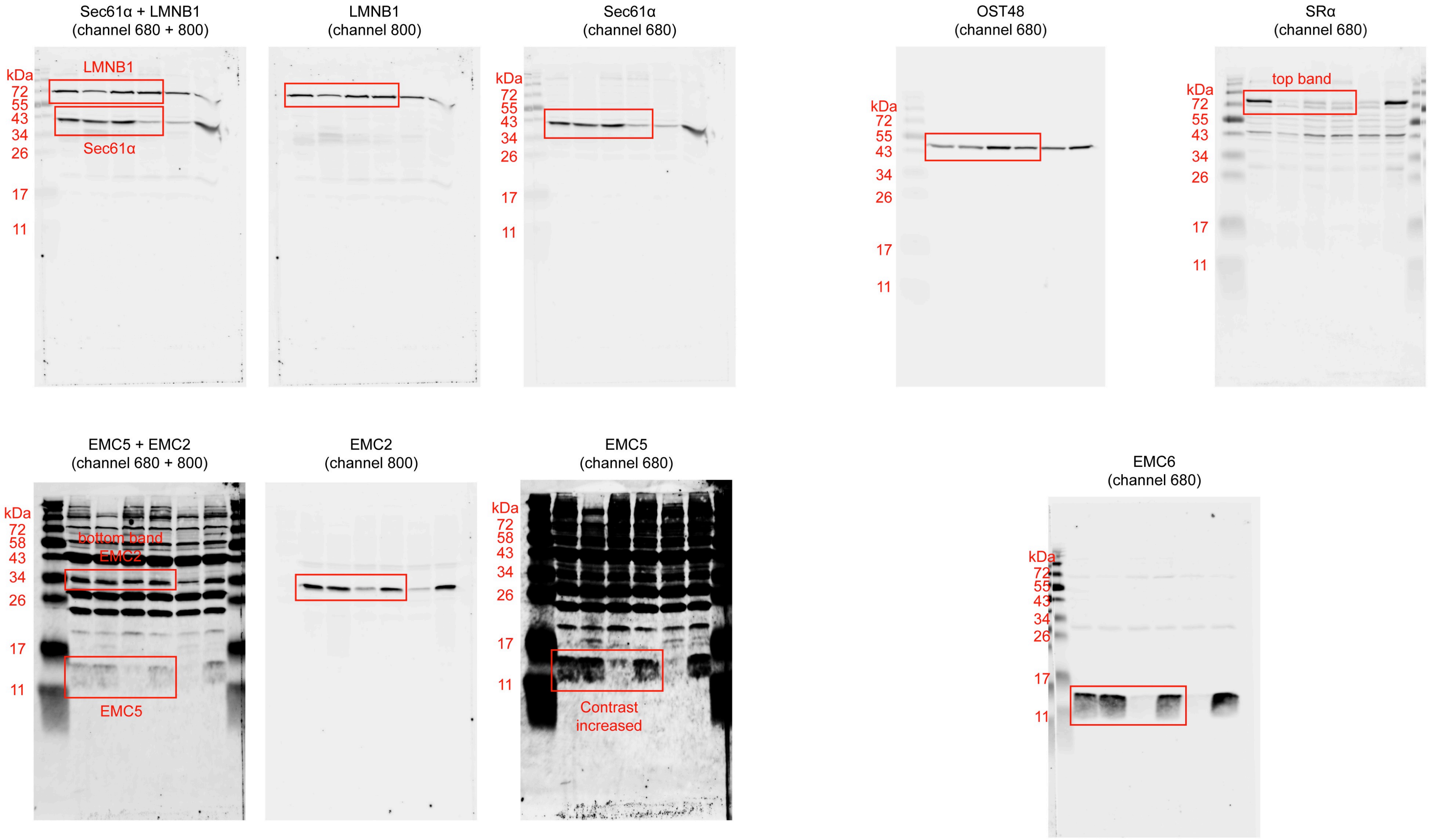

Fig. 6d: Phosphorimages of NT, SRα-kd, SRα+EMC5-kd, SRα+Sec61α-kd insertion assay

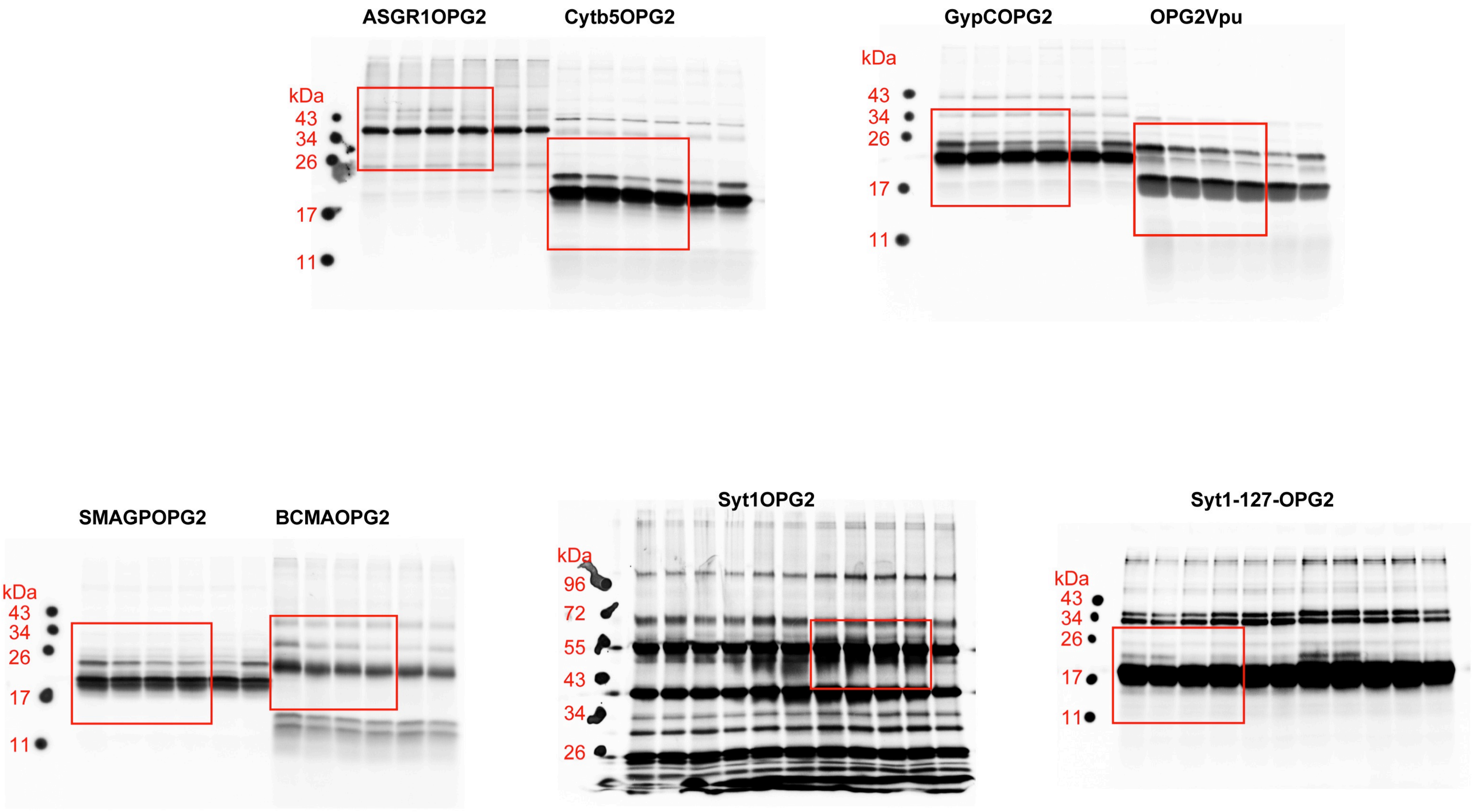

Supplementary Fig. 1: Phosphorimages of +/- Ipom-F insertion assay in microsomes and SP cells

Microsomes

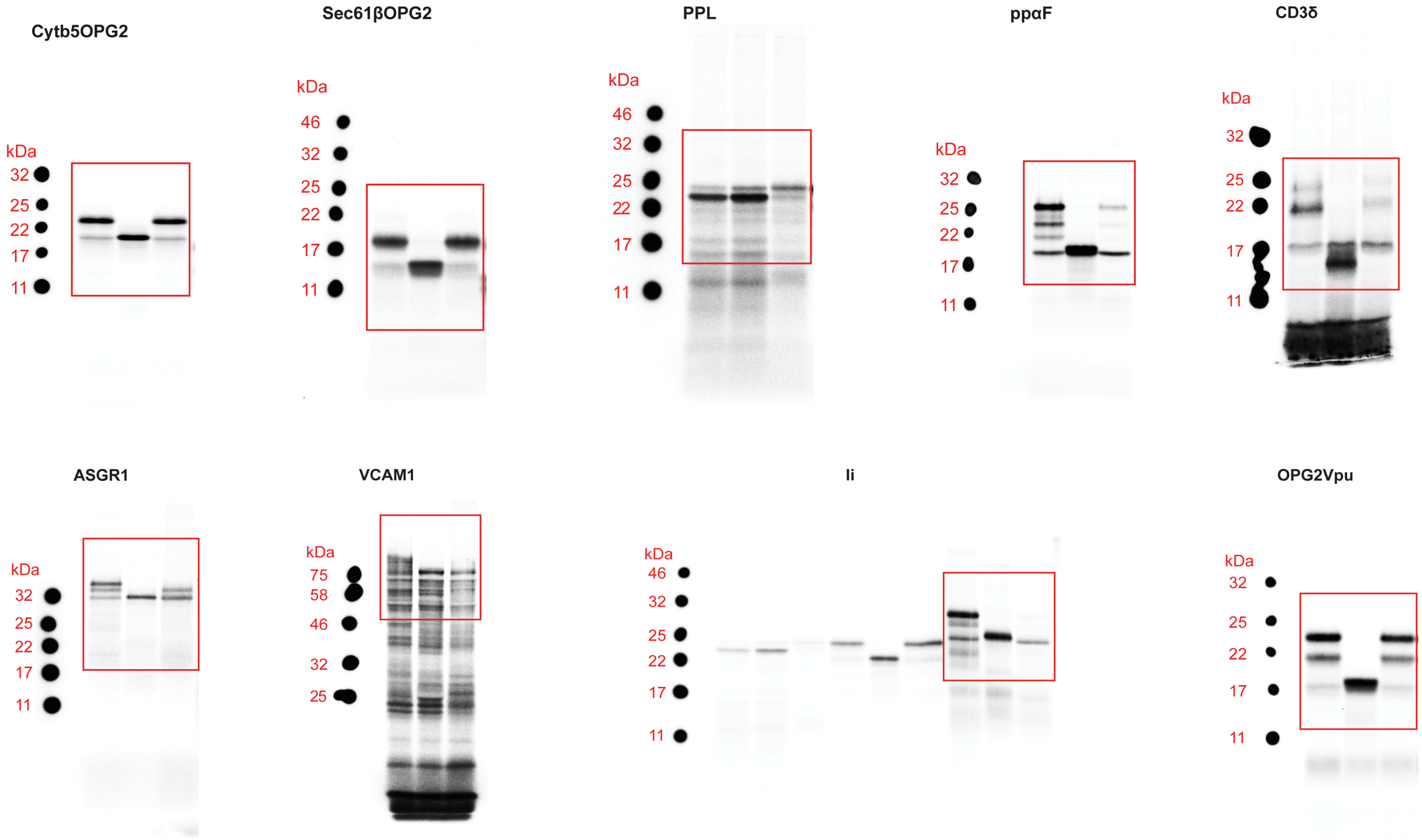

SP Cells

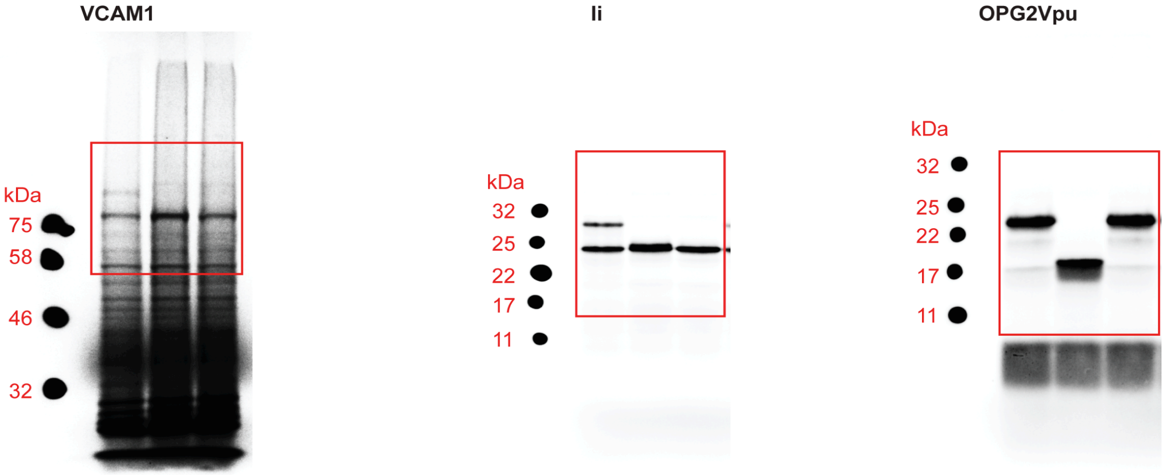

VCAM1
kDa
75
58
46
32
Ii
kDa
32
25
22
17
11
OPG2Vpu
kDa
32
25
22
17
11

Supplementary Fig. 3b: Phosphorimages of +/- Ipom-F insertion assay in microsomes

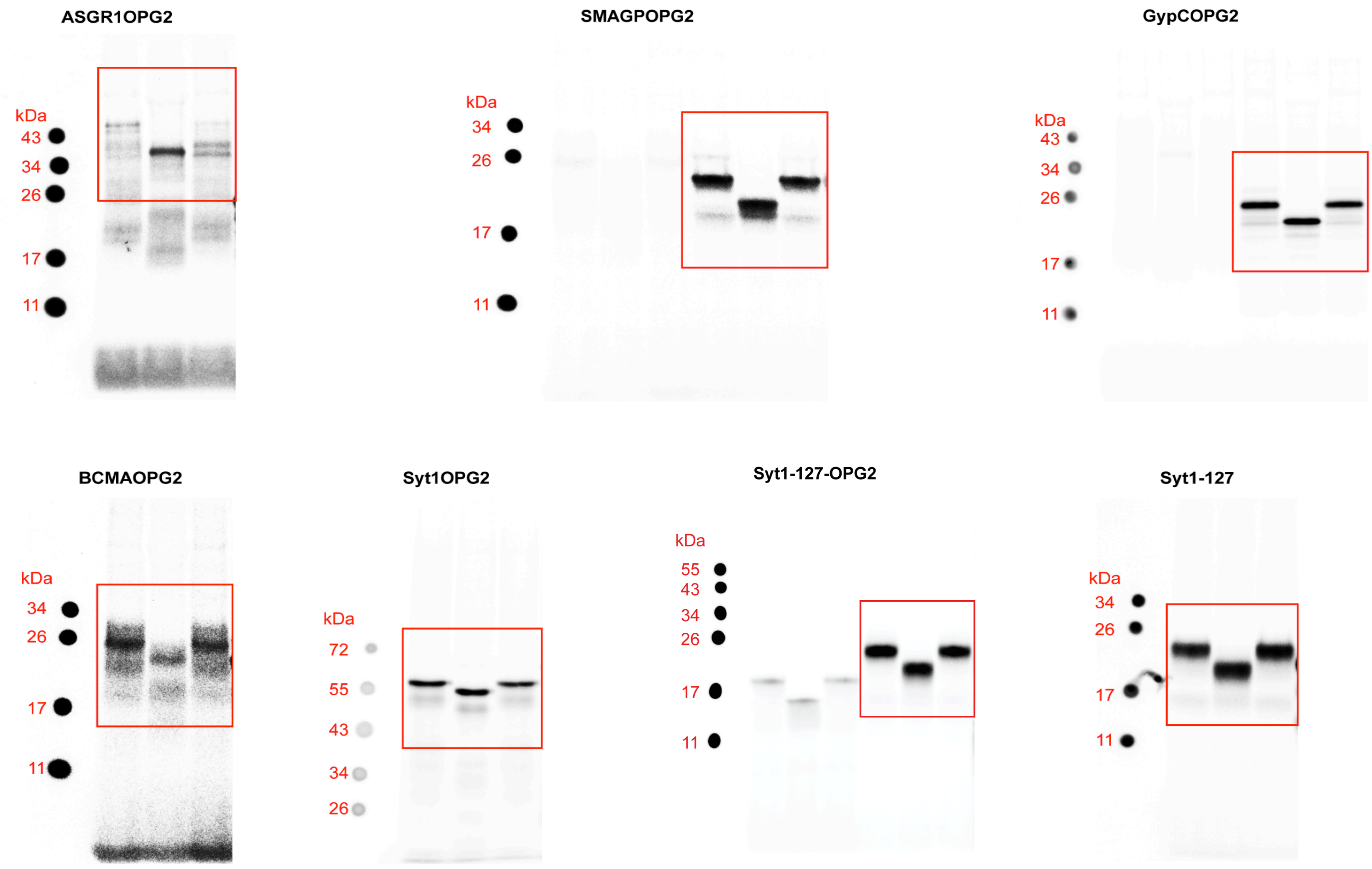

Supplementary Fig. 3c: Phosphorimages to determine the immunoprecipitation efficiency OPG2-tagged TMPs

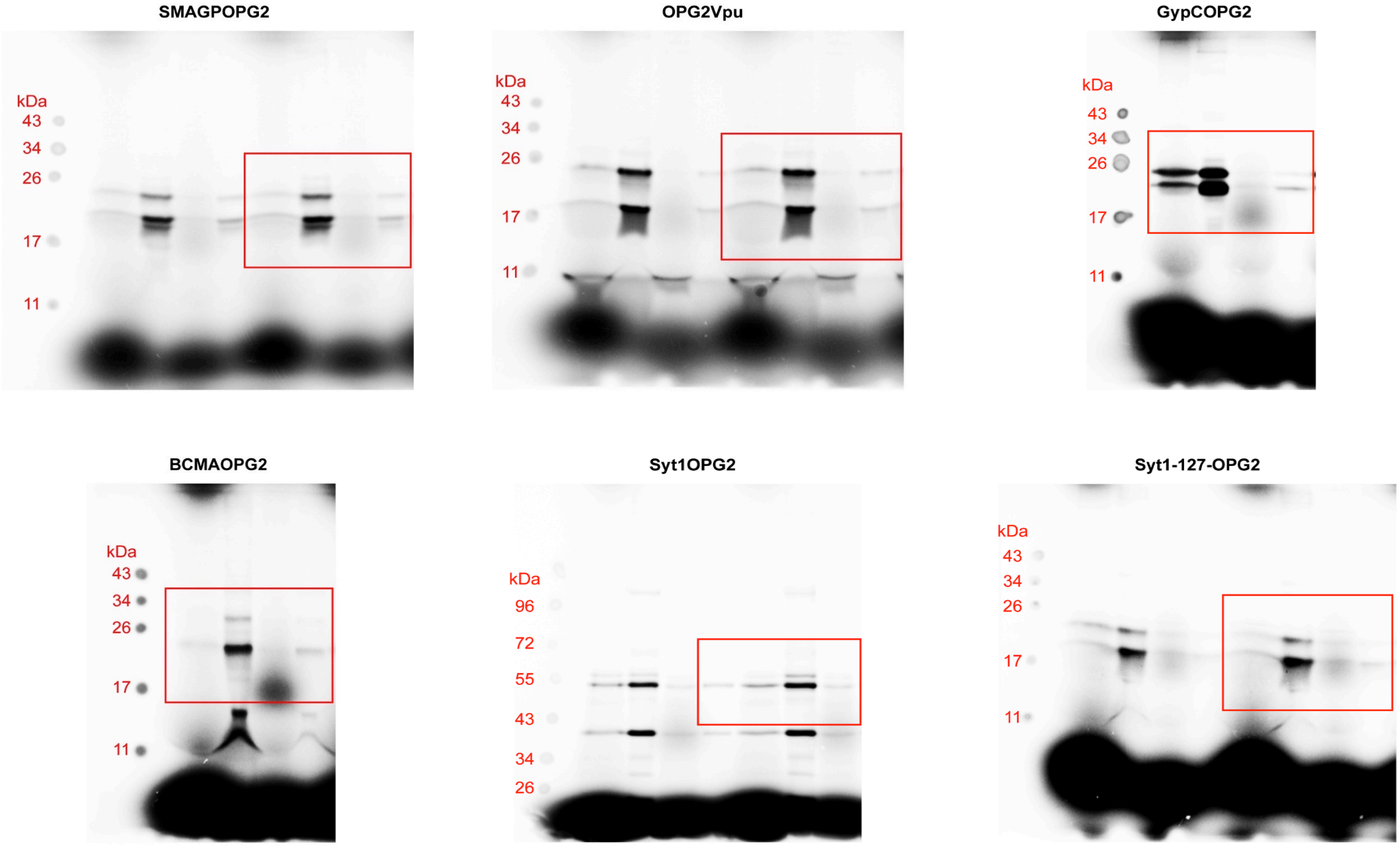

Supplementary Fig. 5b: NT, Sec61α-kd, EMC2-kd, EMC5-kd, Sec61α+EMC2-kd, Sec61α+EMC5-kd, SRα-kd, SRα+EMC5-kd, SRα+Sec61α-kd Western blots

Single knockdowns of Sec61α, EMC2 and EMC5 (lanes 1-4, left panels)

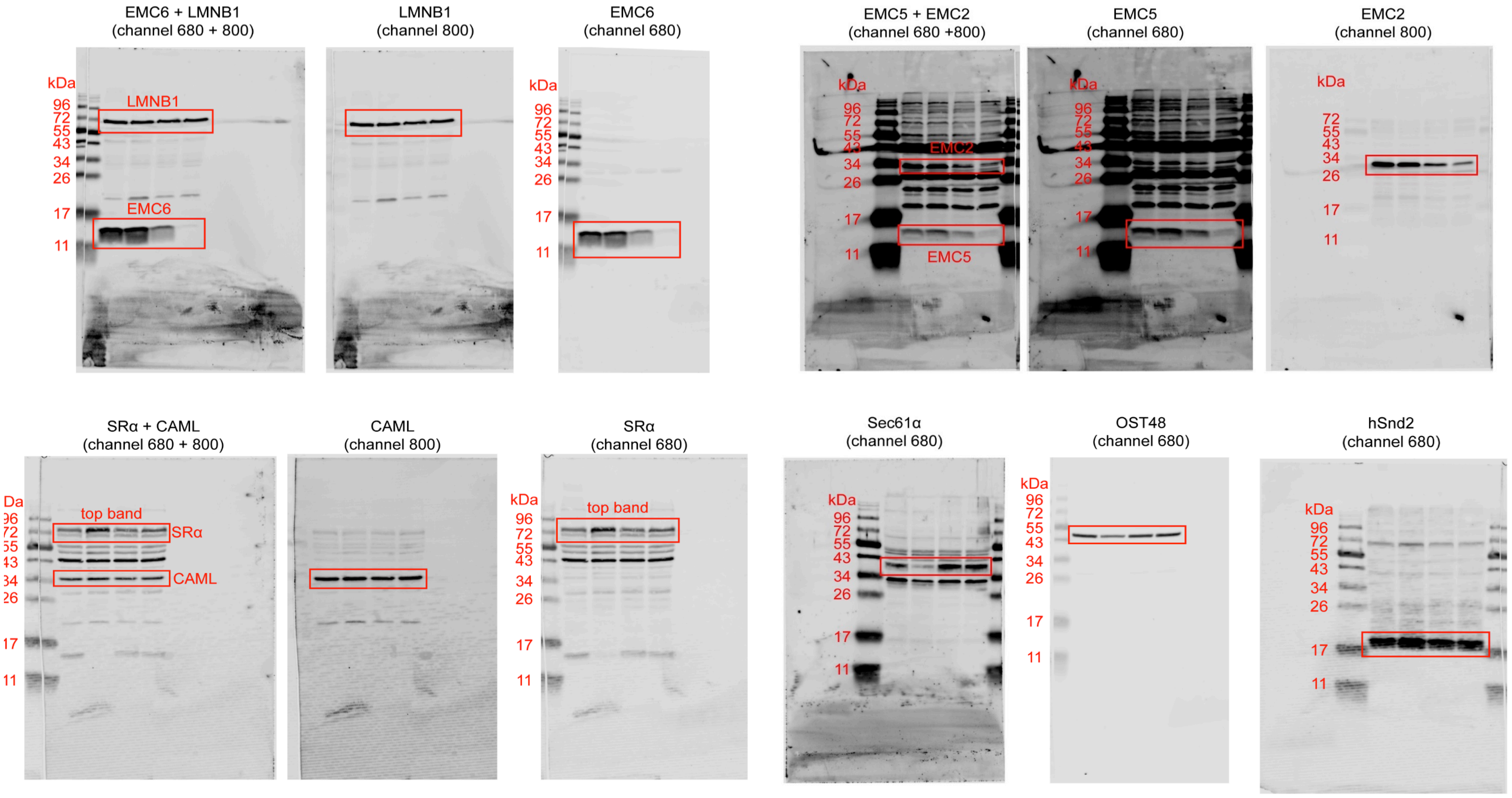

Double knockdowns of Sec61α+EMC2 and Sec61α+EMC5 (lanes 5-7, left panels)

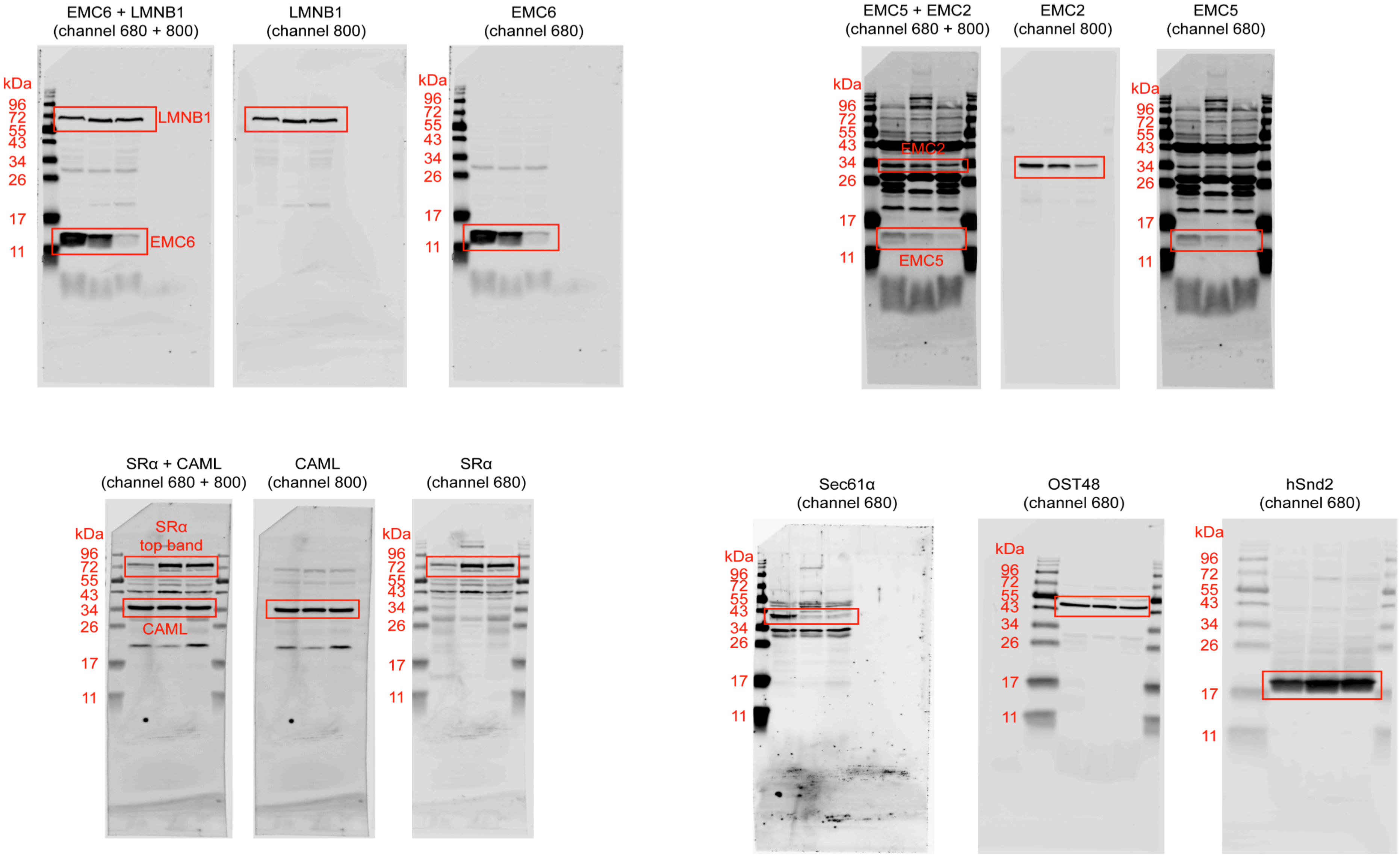

Knockdowns of SRα (lanes 8-9, left panels)

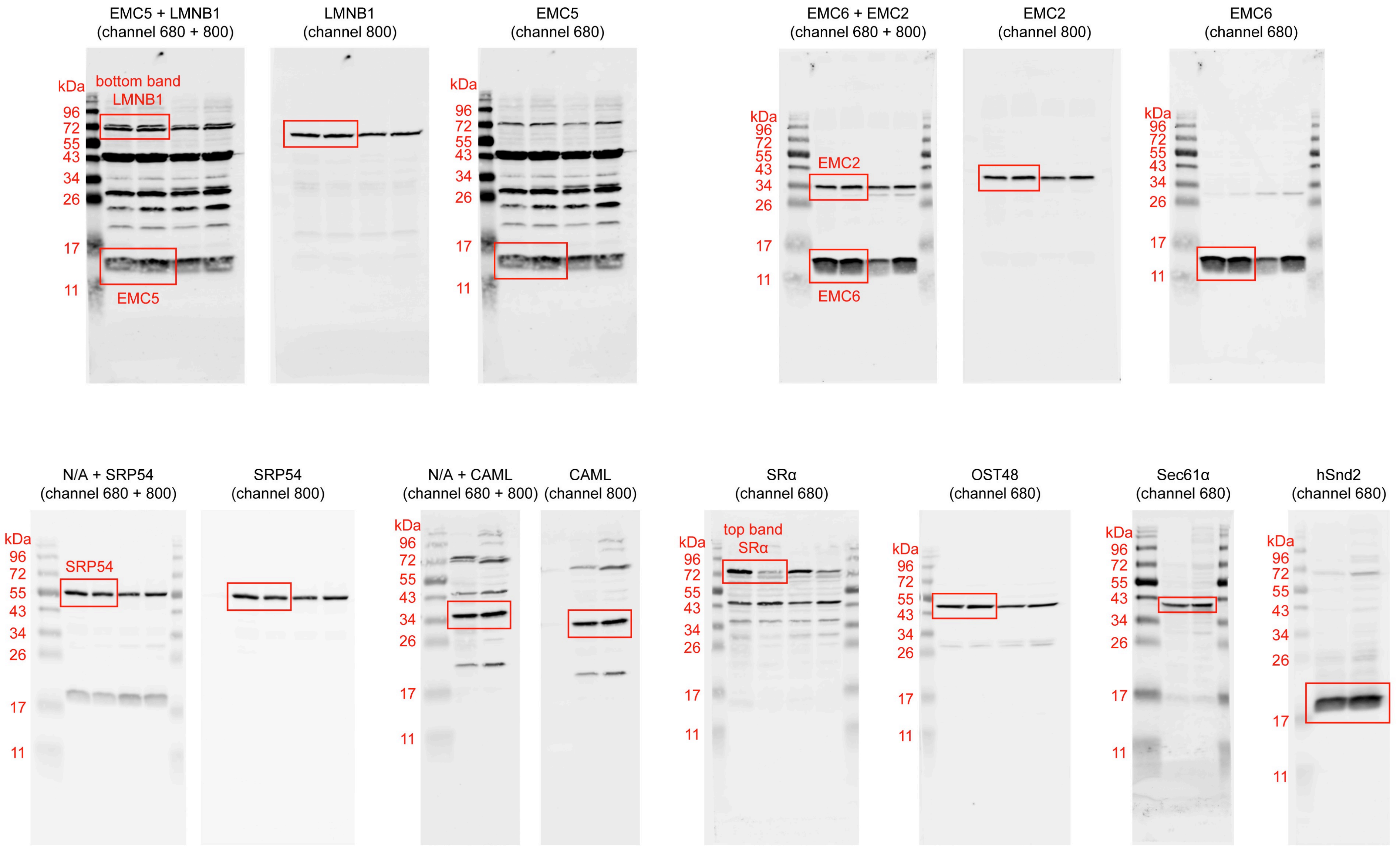

Knockdowns of SRα+EMC5, SRα+Sec61α (lanes 10-12, left panels)

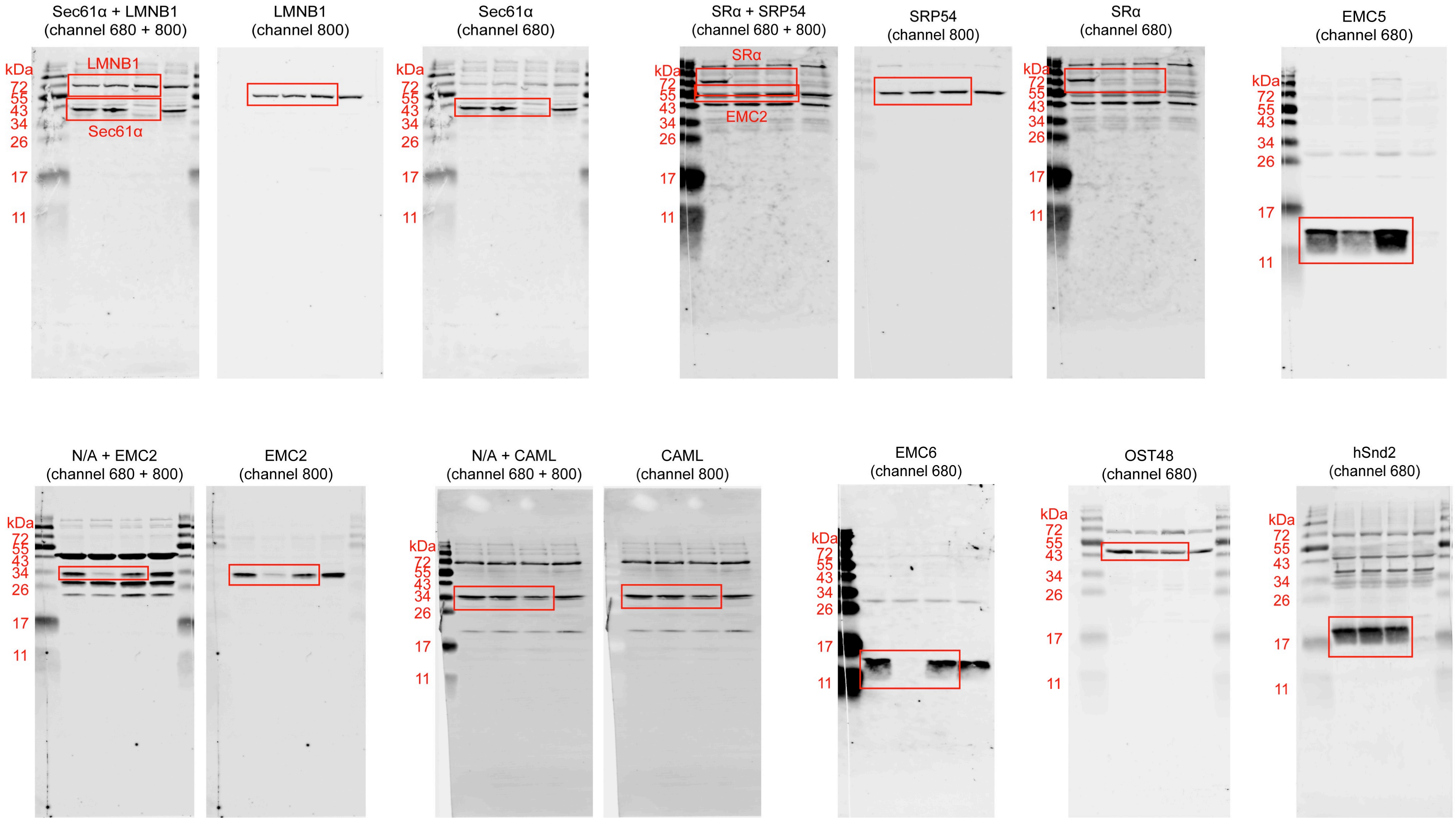

Knockdowns of Sec61α, EMC2, EMC5, Sec61α+EMC5 (lanes 1-4, 7, right panels)

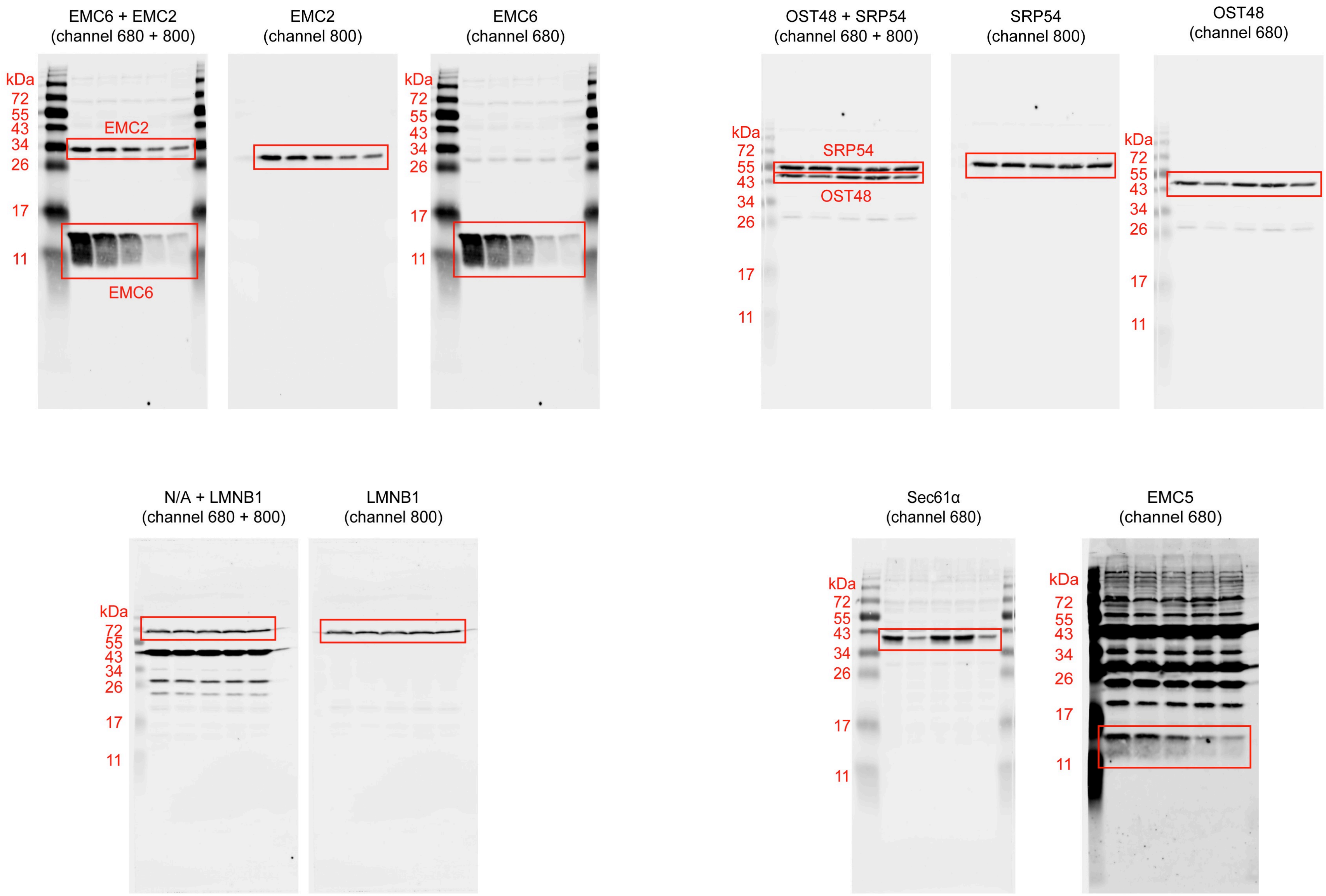

Supplement: Supplementary file 2 — Supplementary Information [file 42003_2021_2363_MOESM2_ESM.pdf]
