# Supplementary material for: An alternative pathway for membrane protein biogenesis at the endoplasmic reticulum
Source: Commun Biol. 2021 Jul 1;4:828. doi: 10.1038/s42003-021-02363-z (PMC8249459; doi:10.1038/s42003-021-02363-z)
Supplement: Supplementary file 3 — Description of Supplementary Files [file 42003_2021_2363_MOESM3_ESM.pdf]

Description of Additional Supplementary Files

File name: Supplementary Data 1
Description: Analysis of type III TMPs.

File name: Supplementary Data 2
Description: Source data for Figures and Supplementary Figures.
