# Supplementary material for: An alternative pathway for membrane protein biogenesis at the endoplasmic reticulum
Source: Commun Biol. 2021 Jul 1;4:828. doi: 10.1038/s42003-021-02363-z (PMC8249459; doi:10.1038/s42003-021-02363-z)
Supplement: Supplementary file 6 — Reporting Summary [file 42003_2021_2363_MOESM6_ESM.pdf]

nature research

Corresponding author(s): Sarah O'Keefe, Stephen High

Last updated by author(s): May 25, 2021

Reporting Summary

Nature Research wishes to improve the reproducibility of the work that we publish. This form provides structure for consistency and transparency in reporting. For further information on Nature Research policies, see our Editorial Policies and the Editorial Policy Checklist.

Statistics

For all statistical analyses, confirm that the following items are present in the figure legend, table legend, main text, or Methods section.

n/a	Confirmed	
☐	☒	The exact sample size (n) for each experimental group/condition, given as a discrete number and unit of measurement
☐	☒	A statement on whether measurements were taken from distinct samples or whether the same sample was measured repeatedly
☐	☒	The statistical test(s) used AND whether they are one- or two-sided<br>*Only common tests should be described solely by name; describe more complex techniques in the Methods section.*
☒	☐	A description of all covariates tested
☒	☐	A description of any assumptions or corrections, such as tests of normality and adjustment for multiple comparisons
☐	☒	A full description of the statistical parameters including central tendency (e.g. means) or other basic estimates (e.g. regression coefficient) AND variation (e.g. standard deviation) or associated estimates of uncertainty (e.g. confidence intervals)
☐	☒	For null hypothesis testing, the test statistic (e.g. F, t, r) with confidence intervals, effect sizes, degrees of freedom and P value noted<br>*Give P values as exact values whenever suitable.*
☒	☐	For Bayesian analysis, information on the choice of priors and Markov chain Monte Carlo settings
☒	☐	For hierarchical and complex designs, identification of the appropriate level for tests and full reporting of outcomes
☒	☐	Estimates of effect sizes (e.g. Cohen's d, Pearson's r), indicating how they were calculated

Our web collection on statistics for biologists contains articles on many of the points above.

Software and code

Policy information about availability of computer code

Data collection	No software was used.
Data analysis	GraphPad Prism v8; Adobe Illustrator 2020; Adobe Photoshop CS4; AIDA Image Analyzer v5.1; Image Studio Lite v.5.2

For manuscripts utilizing custom algorithms or software that are central to the research but not yet described in published literature, software must be made available to editors and reviewers. We strongly encourage code deposition in a community repository (e.g. GitHub). See the Nature Research guidelines for submitting code & software for further information.

Data

Policy information about availability of data

All manuscripts must include a data availability statement. This statement should provide the following information, where applicable:

- Accession codes, unique identifiers, or web links for publicly available datasets
- A list of figures that have associated raw data
- A description of any restrictions on data availability

The authors declare that the data supporting the findings of this study are available within the paper and its supplementary information files.

Field-specific reporting

Life sciences study design

All studies must disclose on these points even when the disclosure is negative.

Sample size	All translation experiments were performed in triplicate. All siRNA treated semi-permeabilised cells that were included in translation experiments were analysed by immunblotting to confirm the efficiency subunit depletion.
Data exclusions	No data was excluded from the analyses.
Replication	Each translation experiment was repeated at least three times using semi-permeabilised cells depleted of ER components via separate siRNA treatments. In all cases, siRNA-depletted semi-permeabilised cells were analysed by immunoblotting for target gene products (at least three times) and non-target gene products (at least two to three times). All attempts at replication were successful.
Randomization	Not relevant to the study.
Blinding	Not relevant to the study.

Reporting for specific materials, systems and methods

We require information from authors about some types of materials, experimental systems and methods used in many studies. Here, indicate whether each material, system or method listed is relevant to your study. If you are not sure if a list item applies to your research, read the appropriate section before selecting a response.

Materials & experimental systems

n/a	Involved in the study	
☐	☒	Antibodies
☐	☒	Eukaryotic cell lines
☒	☐	Palaeontology and archaeology
☒	☐	Animals and other organisms
☒	☐	Human research participants
☒	☐	Clinical data
☒	☐	Dual use research of concern

Methods

n/a	Involved in the study	
☒	☐	ChIP-seq
☒	☐	Flow cytometry
☒	☐	MRI-based neuroimaging

Antibodies

Antibodies used	Primary Antibodies:<br>goat polyclonal anti-LMNB1 (Santa Cruz, sc-6217, clone M-20)<br>guinea pig anti-CAML (Synaptic Systems, 359 004)<br>mouse monoclonal opsin antibody (made in-house)<br>mouse monoclonal TTC35 (EMC2; Santa Cruz, sc-166011, clone D-7)<br>rabbit hSnd2 (provided by Sven Lang, University of Saarland)<br>rabbit SRa (provided by Martin Pool, University of Manchester)<br>rabbit polyclonal MMGT1 (EMC5; Bethyl Laboratories, A305-832-A)<br>rabbit polyclonal transmembrane protein 93/EMC6 (Abcam, ab84902)<br>rabbit polyclonal anti-OST48 (made to order by Invitrogen, Paisley, UK)<br><br>Secondary Antibodies:<br>IRDye 680RD Donkey anti-Goat (LI-COR Biosciences, 926-68074)<br>IRDye 680RD Donkey anti-Rabbit (LI-COR Biosciences, 926-68073)<br>IRDye 800CW Donkey anti-Guinea pig (LI-COR Biosciences, 926-32411)<br>IRDye 800CW Donkey anti-Mouse (LI-COR Biosciences, 926-32212)
Validation	All antibodies were validated by their source company/provider. The specificity of anti-EMC antibodies that were used by us for the first time in this study were checked using cells transfected with EMC subunit siRNAs.

Eukaryotic cell lines

Policy information about cell lines

Cell line source(s)	HeLa cells were as previously published (Roboti et al. 2015, DOI: 10.1242/jcs.166710).
Authentication	HeLa cells were as previously published (Roboti et al. 2015, DOI: 10.1242/jcs.166710).
Mycoplasma contamination	The HeLa cell line used tested negative for mycoplasma contamination.

Commonly misidentified lines<br>(See ICLAC register)	Not applicable.
